# Multiple genomic approaches reveal geographic structure and local selection signals in invasive *Anopheles stephensi* from the Horn of Africa and Yemen

**DOI:** 10.1186/s12864-026-13079-x

**Published:** 2026-07-07

**Authors:** Isuru Gunarathna, Jeanne N. Samake, Ayomikun Aderounmu, Yasser Abdullah Baheshm, Solomon Yared, Dejene Getachew, Said Ali, Elizabeth Waymire, Madison Follis, Avery A. Kaye, Eesha Vasisht, Grace Lloyd, Philip Lavretsky, Sarah Zohdy, Alia Zayed, Tamar Carter

**Affiliations:** 1 Department of Biology, Baylor University, Waco, TX USA; 2 National Malaria Control Program, Ministry of Health, Aden, Yemen; 3 Jigjiga University, Jigjiga, Ethiopia; 4 Department of Applied Biology, Adama Science and Technology University, Adama, Ethiopia; 5 National Malaria Control Program, Ministry of Health Development, Hargeisa, Somaliland; 6 Department of Biological Sciences, University of Texas at El Paso, El Paso, TX USA; 7 Entomology Branch, U.S. Centers for Disease Control and Prevention, Atlanta, GA USA; 8 Faculty of Science, Cairo University, Cairo, Egypt

**Keywords:** *Anopheles stephensi*, Adaptations, Yemen, Ethiopia, Population genomics

## Abstract

**Background:**

*Anopheles stephensi*, an invasive malaria vector expanding across the Horn of Africa, poses a growing public health threat. We applied a multi-faceted genomic approach combining whole genome sequencing (WGS), double-digest restriction-site associated DNA sequencing (ddRAD), and mitochondrial data to resolve population structure and adaptive evolution among *An. stephensi* populations from Ethiopia, Somaliland and Yemen relative to Indian lab-reared colony.

**Results:**

WGS revealed broad-scale differentiation across the autosomal chromosomes, while ddRAD with a larger number of samples (*n* = 402) provided finer resolution of substructure within Ethiopia and Yemen, including sites where heightened malaria has been reported post invasion. Distinct genetic compositions in Sayhut, Yemen, indicate cryptic diversity within *An. stephensi*. WGS-based scans identified selective sweeps in Ethiopian populations at loci encoding homologs of esterases, serine proteases, and lipases, and in Yemeni populations at cytochrome P450 gene clusters.

**Conclusions:**

These results provide insight into *An. stephensi* diversity, particularly for locations where heightened malaria has been reported post-invasion and demonstrate that a combined WGS and ddRAD approach can offer a cost-effective option to resolve population structure and identify adaptive loci in a vector invasion scenario. Integrating these genomic approaches enhances understanding of invasion dynamics, informing surveillance and targeted vector control in urbanizing regions.

**Supplementary Information:**

The online version contains supplementary material available at https://doi.org/10.1186/s12864-026-13079-x.

## Introduction

Malaria remains a significant global health challenge, with an estimated 263 million cases and 597, 000 deaths worldwide in 2023 alone [1]. Despite sustained global efforts toward malaria vector control, the emergence and spread of invasive vector species pose significant threats to public health. *Anopheles stephensi*, originally native to South Asia and the Middle East, has rapidly expanded its geographical range over the past decade, establishing populations in regions of Africa and the Arabian Peninsula previously unaffected by this species (2–4]. Its tendency to thrive in urban environments, particularly the ability to utilize artificial water containers as larval habitats, contrast sharply with native *Anopheles* species predominantly breeding in rural, agricultural settings [5]. This characteristic significantly increases malaria transmission potential in urban and peri-urban settings, exacerbating public health risks in invaded regions[6].

Recent genomic studies in Yemen and the Horn of Africa (HoA) have highlighted substantial genetic differentiation among *An. stephensi* populations, suggesting multiple invasion events and potential sources [7–9]. For example, genomic analyses demonstrated clear population structuring within Ethiopia and Yemen, reflecting sequential founder events possibly influenced by geographic isolation and human-mediated dispersal [10, 11]. Genetic connectivity analyses revealed the Dire Dawa population in Ethiopia as a central hub, suggesting its role as a major hub from which sequential founder events occurred in the rest of the eastern Ethiopian region [10].

While previous genomic studies [10–12] employing ddRAD and mitochondrial data have provided important insights into the population dynamics of invasive *An. stephensi*, the genomic architecture of this species may influence how effectively reduced representation approaches capture informative variation. Because the density of informative polymorphic sites between restriction enzyme cut sites can vary across the *An. stephensi* genome, it is important to evaluate how well ddRAD data captures population structure and signals of selection relative to whole-genome data [13]. Further, ddRAD sequencing offers a cost-effective and efficient approach for genotyping, providing sufficient genomic coverage and accurate variant calling comparable to WGS, while requiring less sequencing effort and resources, making it particularly advantageous for population genomic studies in non-model organisms [14]. Conversely, mitochondrial data inform on historical maternal lineage diversification but offer limited resolution for detecting contemporary population structure due to lower variant density [15].

Assessing population structure and understanding genomic adaptations are crucial in the context of the *An. stephensi* invasion, as these insights help identify the molecular mechanisms driving successful colonization and persistence in novel environments[16]. Population genomic analyses reveal how distinct populations respond to localized selective pressures such as insecticides, urbanization, and climatic variation [17, 18]. Moreover, deciphering these adaptations provides broader insights into invasion trajectories, facilitating the prediction of potential geographic spread and informing region-specific surveillance and targeted vector control strategies.

The objective of this study is to characterize population structure and adaptive evolution in invasive *An. stephensi* populations from Ethiopia and Yemen. These regions represent key areas of ongoing invasion, where populations are likely experiencing novel environmental and anthropogenic selective pressures. We additionally evaluate how well ddRAD markers capture these biological patterns to assess the utility of reduced-representation approaches for surveillance in this species. Field populations from Ethiopia and Yemen were selected to capture key regions of the invasive range, including areas with reported increases in malaria transmission [19]. The Indian laboratory colony was included as a reference dataset to provide comparative context for divergence patterns, rather than as a direct proxy for wild ancestral populations. By leveraging a combined genomic approach, we aim to provide nuanced insights into the population structure, genetic differentiation, and adaptive evolution of this invasive malaria vector. Ultimately, this research seeks to address critical knowledge gaps regarding the genomic factors underpinning the recent invasion dynamics of *An. stephensi*. It also contributes vital information for targeted vector control strategies and surveillance measures to halt further spread and mitigate malaria transmission risks in affected regions.

## Methods

### Sample collection, sequencing and variant calling

**Fig. 1:**
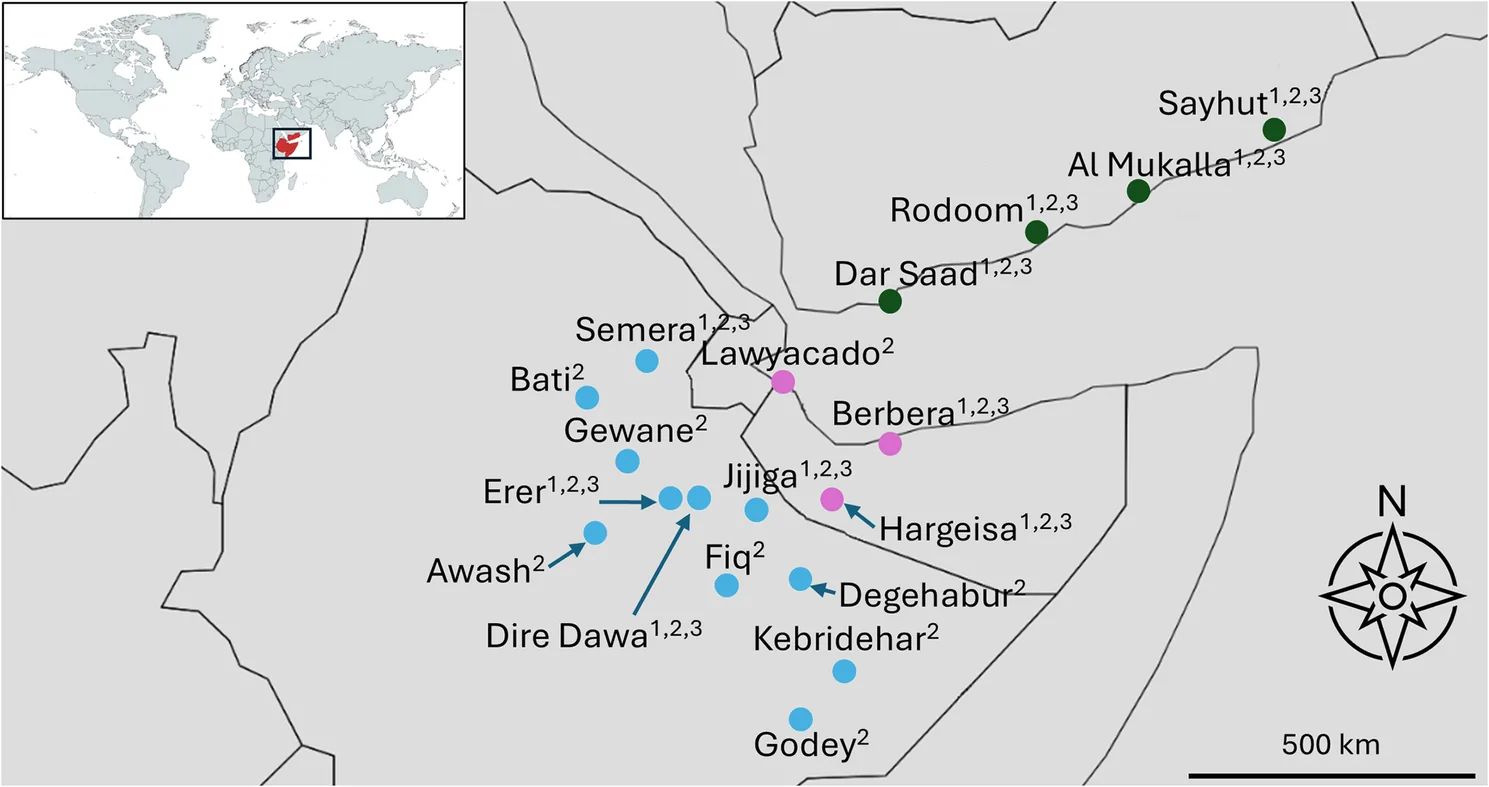
Geographic distribution of *Anopheles stephensi* sampling sites across Ethiopia, Somalia and Yemen. Colored circles denote country of origin (light blue = Ethiopia; pink = Somaliland; dark green = Yemen), and each site name is annotated with superscript codes indicating which genomic data were generated: ¹ = WGS, ² = ddRAD, ³ = mitochondrial. Inset (upper left) shows the study region in its continental context. Map source for the python script - https://raw.githubusercontent.com/johan/world.geo.json/master/countries.geo.json)

Samples of *An. stephensi* were collected from sites across Ethiopia, Yemen and Somaliland between 2018 and 2023 (Fig. 1). The set of samples collected from each country were considered as the populations in the analyses (for more details on number of samples and collection sites, see Supplementary Table 1). The set of Indian wild-type laboratory strain genomes (*n* = 20) were taken from a previously published study [20] where they collected original larvae samples in 2018 from Bangalore and Mangalore cities in Karnataka state in India. Wild adult mosquitoes were preserved on silica gel and transported to Baylor University at Waco, Texas (Tx), United States of America for genomic DNA extraction using the QIAGEN DNeasy Blood & Tissue Kit. Whole-genome sequencing (WGS) was performed on the Illumina NovaSeq platform, followed by quality control (FastQC), trimming (Trimmomatic), and alignment to the *An. stephensi* reference genome using BWA-MEM [21–23]. Variant calling was conducted with GATK HaplotypeCaller and GenotypeGVCFs, and SNPs were filtered according to GATK best practices [24]. For mitochondrial genomes, two approaches were used: [1] PCR amplification of targeted regions followed by Sanger sequencing and alignment to the reference mitogenome, and [2] extraction from WGS data via reference-based mapping and variant calling using BCFtools and SAMtools.

For ddRAD-seq, larvae collected from four sites in Yemen and reared, morphologically identified, before sending to be processed at Baylor University, Tx, USA. DNA was digested with SbfI and EcoRI, then barcoded and sequenced using the Illumina HiSeq platform. Reads were demultiplexed, trimmed, and aligned to the reference genome using BWA, with variant calling performed using BCFtools. The ddRAD sequencing targets genomic regions flanking restriction enzyme cut sites, resulting in a reduced representation dataset with markers distributed according to the underlying distribution of restriction sites across the genome. Only loci containing high confidence polymorphic sites after filtering were retained for downstream analyses. High-confidence variants for all datasets were filtered based on read depth, allele frequency, and base quality for downstream population genomic analyses [10, 13]. For a more detailed description of sample collection, sequencing and variant calling please read the supplementary methods.

In this study we performed population structure analyses using four variant datasets: [1] WGS variants [2], ddRAD variants [3], WGS ddRAD intersect variants, and [4] mitochondrial variants (for number of samples see Supplementary Table 1). The ddRAD based analyses were used to evaluate how well reduced representation markers capture the biological patterns observed in WGS data. A combination of complementary population genomic approaches, including PCA, ADMIXTURE, phylogenetic inference, and window-based summary statistics (H12 and FST), was used to capture patterns of population structure and identify candidate regions under selection. The analyses to detect signals of selection and fixation were only performed on the autosomal chromosome-specific variants from WGS data.

### Principal Component Analysis (PCA)

Principal component analyses were performed on all four data sets to investigate population structure. All four PCAs were conducted using PLINK v1.9 [25, 26]. Independent SNPs were pruned using a sliding window approach, and the resulting variants were used to compute principal components. In linkage pruning step, a sliding window with a width of 50 variants and a step size of 10 variants with R^2^ threshold set to < 0.1 was used for whole genome sequence data and the WGS ddRAD intersection data. For the original ddRAD data set linkage pruning R^2^ threshold was set to < 0.3 to make it less stringent due to the nature of the ddRAD data. Separate PCAs were performed for biallelic phased variants from autosomal chromosomes for whole genome sequence data and ddRAD data. For mitochondrial variants we did not perform the linkage pruning due to the high linkage observed among mitochondrial variants. PCAs were visualized using ggplot2 in R, separately for the autosomal chromosomes, as well as for mitochondrial and ddRAD datasets. PCAs were recreated for WGS dataset and WGS ddRAD intersection data set without including Indian samples. The original ddRAD dataset did not have the variants called with the Indian samples, therefore a single PCA was performed without Indian samples.

### Phylogenetic analysis

Population genomic relationships and evolutionary history among *An. stephensi* populations were inferred using TreeMix v1.13, a method based on allele frequency correlations that is specifically suited for sexually recombining species [27]. Analyses were conducted using single-nucleotide polymorphism (SNP) datasets derived from whole-genome sequencing (WGS), WGS ddRAD intersect, and ddRAD variants. For each dataset, filtered biallelic variants were first processed using PLINK (v1.9) to calculate stratified allele frequencies across defined population clusters. To mitigate the confounding effects of linkage disequilibrium (LD) inherent in genome-wide data, variants were grouped into physical blocks of 100 SNPs (k = 100) using block jackknifing. Maximum-likelihood (ML) baseline population graphs were constructed under a strict non-admixture model (m = 0). To assess node support and topology stability, a majority-rule consensus approach was implemented by executing 1,000 independent bootstrap replicates, resampling blocks of 100 SNPs with replacement. In the absence of an unambiguous biological outgroup, all resulting trees were left unrooted. The 1,000 bootstrapped trees were subsequently summarized into a final majority-rule consensus tree (*p* = 0.5) using the consensus function in the R package ape. Separate population trees were constructed for the WGS, WGS ddRAD intersect, and ddRAD datasets. To better resolve fine-scale relationships among geographic populations, separate analyses were performed for the WGS and WGS ddRAD intersect datasets by excluding Indian laboratory-reared samples (the ddRAD dataset natively excluded these samples). Final consensus trees were visualized and annotated using FigTree v1.4.4, with leaf nodes representing distinct population clusters color-coded according to geographic origin.

### Admixture analysis

Genetic ancestry proportions were estimated using ADMIXTURE v1.3 [28]. This analysis was performed on all four data sets except for the mitochondrial genome variants dataset. In order to determine the optimal number of population clusters (K), cross-validations were performed with K = 2–10. Separate analyses were run for all populations, and the admixture proportions were plotted for each sample in each data set using a matplotlib python library. Prior to ADMIXTURE analyses of WGS and WGS ddRAD intersect datasets, SNPs with minor allele Frequency lower than 0.01 and Genotype missingness per SNP greater than 10%, Individual missingness > 10% (--maf 0.01, --geno 0.1, --mind 0.1) filtered using PLINK. SNPs with a linkage R^2^ > 0.2 were removed using PLINK with a sliding window of 50 variants with a step size of 5 variants (--indep-pairwise 50 5 0.2). For the ddRAD only data set thresholds were set to exclude variants with minor allele frequency lower than 0.01 and Genotype missingness per SNP greater than 30% and Individual missingness > 10% (--maf 0.01 --geno 0.1 --mind 0.3). ddRAD SNPs with a linkage R^2^ > 0.3 were removed using PLINK with a sliding window of 50 variants with a step size of 5 variants (--indep-pairwise 50 5 0.3).

### Selective sweep detection with H12

In order to detect recent positive selection, the Garud’s H12 statistic was computed using the Python library scikit-allel [29]. H12 statistics identify selective sweeps based on haplotype homozygosity. H12 values were calculated only for the WGS dataset across Chromosomes 2 and 3 using non-overlapping 100 kb windows, after variants were phased using Beagle v5.4, and haplotypes were extracted for each window [30]. The 99th percentile of H12 values was used as a threshold to identify regions of interest. Genes within high-H12 windows were annotated using the *An. stephensi* genome annotations from National Center for Biotechnology Information (NCBI) as well as other literature. Alongside H12 calculation, we estimated per-individual, and per-chromosome runs of homozygosity (ROH) using the function implemented in scikit-allel python library, “allel.roh_mhmm()”. The resulting data were aggregated to derive summaries for each population, specifically the proportion of individuals in the population with of ROH segments for given window and the proportion of the genome covered by ROH (filtered out segments with a proportion of individuals in population > 0.5 from selective sweep analysis). To focus specifically on recent inbreeding events, we retained only those ROH segments not exceeding 100 kb in length [11] in selection sweep scan.

### Genome-wide differentiation (F_ST_)

It is important to identify genomic features that differ among the three populations (Indian laboratory-reared, Yemen, and Ethiopia). Therefore, we calculate genome-wide F_ST_ using the WGS dataset. The genomic regions with outlier statistics (F_ST_ > 99th percentile) may correspond to loci under divergent selection in different environments. Pairwise Weir and Cockerham’s F_ST_ values were calculated across the genome using VCFtools, employing 100 kb overlapping windows with 50 kb step size [31]. Comparisons included Ethiopia vs. India, Yemen vs. India, Ethiopia vs. Yemen, and combined Ethiopia/Yemen vs. India. Peaks above the 99th percentile were considered regions of elevated differentiation. The overlap between high F_ST_ and high H12 windows was used to identify putative adaptive loci.

### Functional inference of fixed genes under selection

Genes located within overlapping high H12 and high F_ST_ windows were extracted and functionally annotated based on homology to characterized genes in *An. gambiae*, *Drosophila melanogaster*, and other insects using NCBI and the VectorBase annotation databases. Functional relevance was determined through literature review of gene functions related to insecticide resistance, immune response, and stress tolerance.

## Results

### Principal component analysis of *Anopheles stephensi* populations

**Fig. 2:**
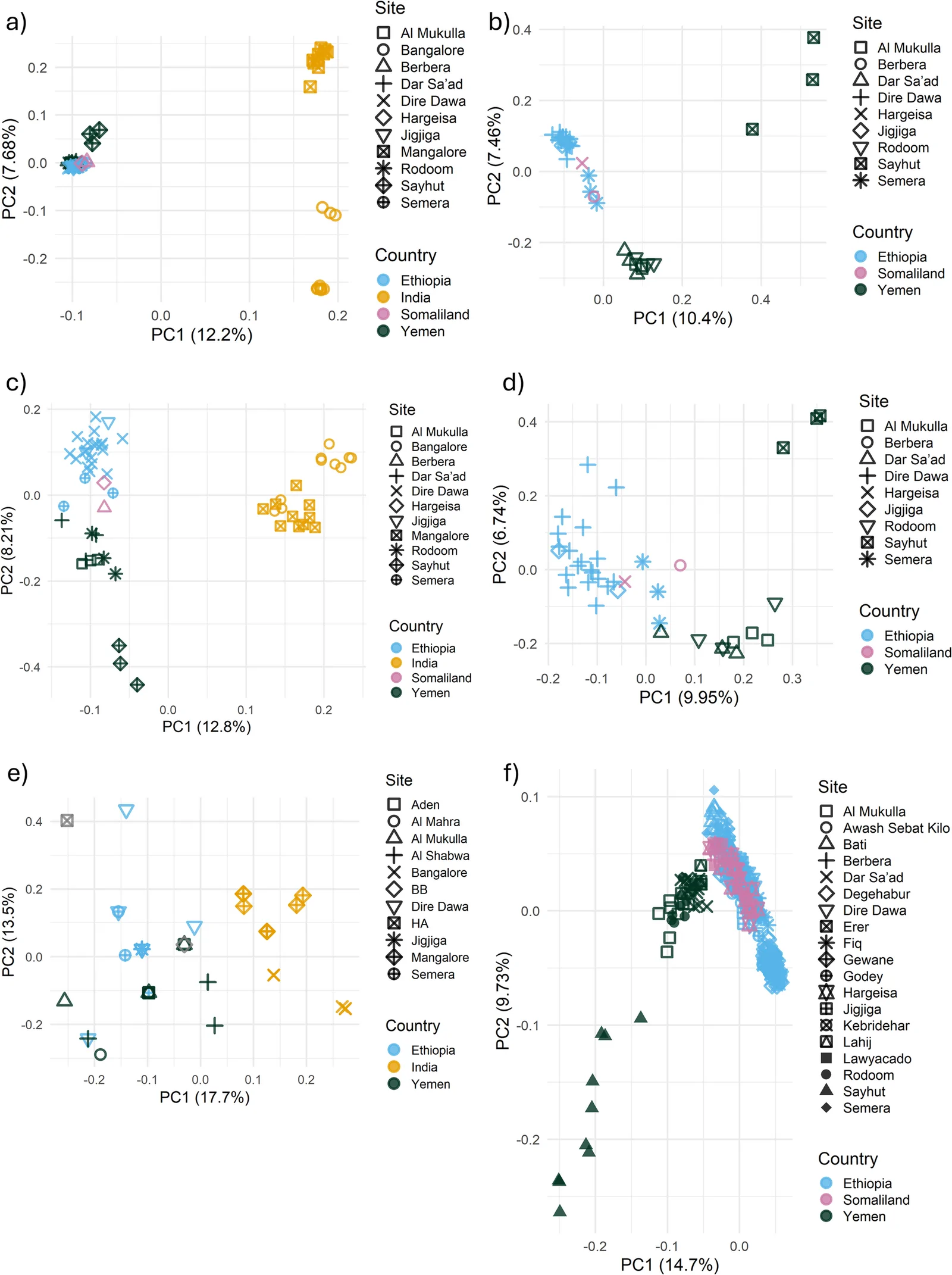
Principal component analysis (PCA) of *An. stephensi* populations from Ethiopia, Somaliland, Yemen, and a laboratory-reared colony from India. Panel (**a**) and (**b**) shows the clustering of samples based on the autosomal variants generated from WGS data with and without Indian samples. Panel (**c**) and (**d**) shows the clustering of samples based on the WGS ddRAD intersect data with and without Indian samples. Panel (**e**) shows the cluster separation of samples based on variants from mitochondrial genomes. Panel (**f**) shows the cluster separation of samples based on autosomal variants generated solely from ddRAD data. The first principal component (PC1) and second principal component (PC2) are shown on the x- and y-axes, respectively, with the percentage of variance explained indicated in parentheses. Ethiopian and Somaliland populations show partial overlap, suggesting some genetic similarity, whereas the Indian lab colony and Yemen samples form a distinct cluster in all analyses

To assess the genetic differentiation among *An. stephensi* populations from Ethiopia, Somaliland, Yemen, and a laboratory-reared colony from India, we conducted PCA using autosomal chromosome-specific variants from WGS data (10,462,700), variants from ddRAD data (5747), WGS ddRAD intersect (4882) and mitochondrial data (62) (Fig. 2). The PCA results revealed distinct clustering patterns, indicating clear genetic structure among four countries and even among the regions in India and Yemen.

The autosomal variants from WGS data have clearly separated the Ethiopian, Somaliland and Yemeni samples from Indian lab reared colony samples along the PC1 axis. However, with Indian samples present in the data set, the separation between the Horn of Africa (HOA) and Yemen is not notable except for the Sayhut samples (Easternmost samples from Yemen). In WGS PCA biplot without Indian samples (panel b Fig. 2) the separation between HOA and Yemen is prominent and samples from Sayhut have shifted away from both HOA and other Yemen samples suggesting distinct genetic compositions. The two Somaliland samples have clustered together with the Semera samples from Ethiopia which are the samples closest to the Yemen samples cluster.

In the two PCA biplots (Fig. 2, panels c and d) based on the WGS ddRAD intersect dataset also have captured the cluster separations in WGS PCA biplots. It is important to note that these variants have captured the cluster separation among HOA and Yemen samples more clearly compared to the cluster separation between the two sites in Indian lab reared colony (Fig. 2, panel c) which was prominent in WGS PCA biplots (panel a and b). The PCA biplot based on the variants from ddRAD data also captured the distinction between HOA samples and Yemen samples and it has confirmed the observation of Sayhut samples being separated from the rest of the Yemen samples (Fig. 2, panel f).

There were multiple overlapping samples in the PCA biplots based on mitochondrial genome variants indicating the lack of information. This could be due to the low number of variants (62) in mitochondrial genome data.

### Population structure and evolutionary relationships via treeMix

**Fig. 3:**
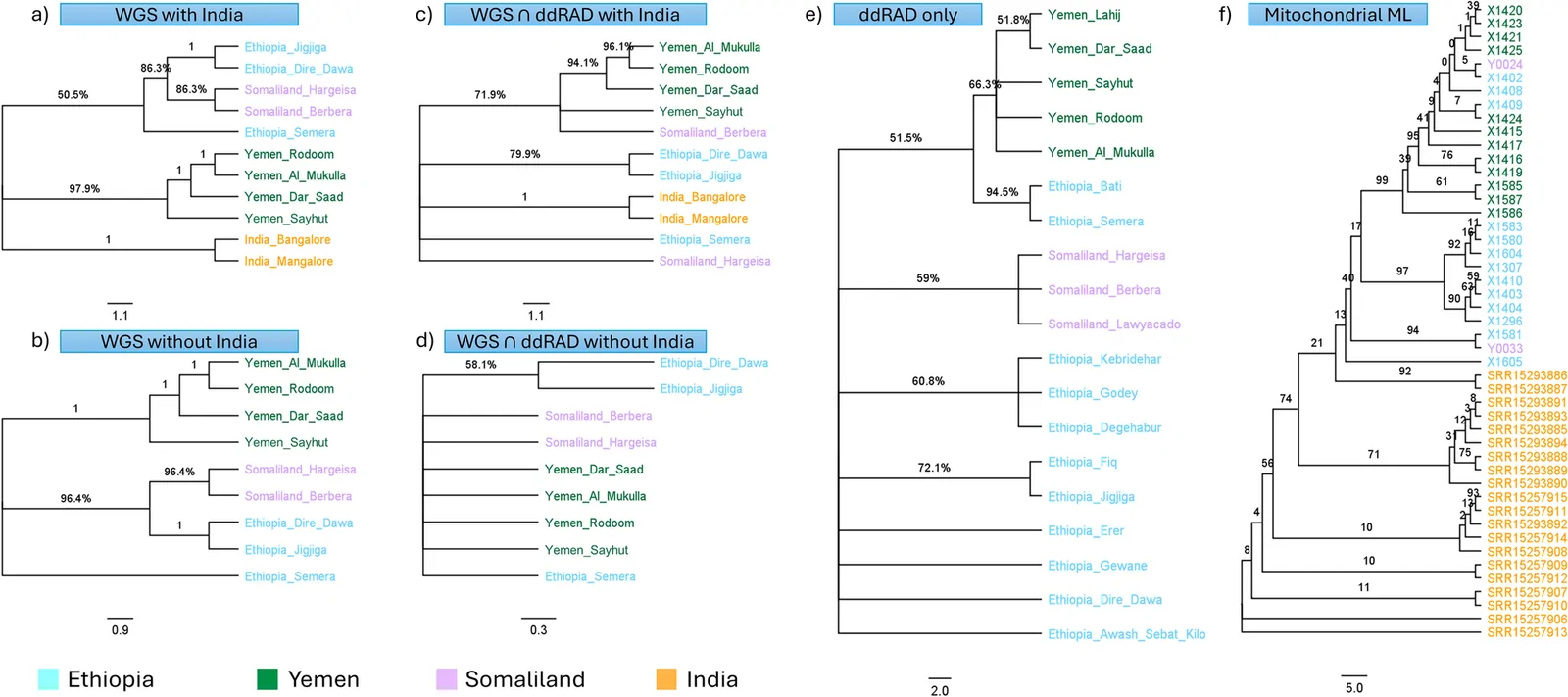
Population genetic structure and evolutionary relationships of *An. stephensi*. Unrooted TreeMix population graphs (m = 0) derived from nuclear variants are shown for whole-genome sequencing (WGS) including Indian laboratory colonies (**a**), WGS excluding India (**b**), the WGS ddRAD intersect dataset including India (**c**), the WGS ddRAD intersect dataset excluding India (**d**), and the ddRAD only dataset (**e**). Population graphs were constructed using genome-wide allele frequency correlations, with linkage disequilibrium accounted for by block jackknifing (k = 100). Node support for nuclear graphs represents the fraction of 1,000 independent bootstrap replicates supporting the topology. Individual-level Maximum Likelihood (ML) phylogenetic tree (**f**) constructed from haploid mitochondrial genome variants under the GTRGAMMA model of nucleotide substitution, with node support assessed via rapid bootstrapping (autoMRE criterion). Horizontal branch lengths for the nuclear population graphs are proportional to the amount of genetic drift experienced by each population, whereas mitochondrial branch lengths correspond to the number of nucleotide substitutions per site. Leaf nodes are color-coded by geographic region of origin: Ethiopia (light blue), Yemen (green), Somaliland (purple), and India (orange)

To resolve the evolutionary relationships and genetic divergence among *An. stephensi* populations from Ethiopia, Somaliland, Yemen, and India, we constructed unrooted maximum-likelihood population trees using TreeMix (m = 0) for the nuclear datasets (WGS, WGS ddRAD intersect, and ddRAD only) and a standard ML tree for the haploid mitochondrial genomes (Fig. 3). Node support was assessed using bootstrap resampling (1,000 replicates for TreeMix and autoMRE for mitochondrial data), with values indicated at internal nodes. Across all nuclear datasets, the recovered topologies strongly reflected geographic structure, mirroring the patterns observed in the PCA and individual-level clustering analyses. In the autosomal chromosome wide WGS population tree including Indian samples (Fig. 3 - a, WGS with India), the Indian laboratory colonies (Bangalore and Mangalore) formed a highly divergent, long-branched clade, consistent with long-term laboratory isolation and pronounced genetic drift. The invasive populations clustered cleanly by geographic region. Notably, the Ethiopian Semera population occupied a position basal to the Yemen clade, rather than clustering with the other Horn of Africa populations (Jigjiga and Dire Dawa), hinting at complex invasion dynamics or shared historical ancestry between Semera and Yemen. When the Indian samples evolved under laboratory conditions, were excluded (Fig. 3 - b, WGS without India), resolution among the field populations improved. The Somaliland populations (Hargeisa and Berbera) clustered together with strong support (96.4%) and shared a clear split with the remaining Ethiopian sites. The population tree based on the WGS ddRAD intersect dataset including India (Fig. 3 - c, WGS ∩ ddRAD with India) recapitulated the WGS topology to a certain extent, maintaining the separation of the Indian. However, when Indian samples were excluded (Fig. 3 - d, WGS ∩ ddRAD without India), the intersect dataset which captures fewer genome wide SNPs exhibited a noticeable collapse in internal node support (58.1% for the primary Ethiopian split), rendering the relationship between the Somaliland and Yemeni clusters less distinct. The ddRAD only dataset provided the highest geographic sampling density across Ethiopia (Fig. 3 - e, ddRAD only). The unrooted TreeMix graph resolved a clear, well-supported split (94.5%) between a northern cluster (consisting of all Yemeni populations alongside Ethiopian Bati and Semera) and the remaining East African populations. Within the Horn of Africa group, the Somaliland populations formed a distinct, moderately supported sub-clade (59%), while the remaining Ethiopian localities (e.g., Kebridehar, Godey, Degehabur, Fiq, and Jigjiga) exhibited fine-scale regional structuring, though several internal nodes showed lower bootstrap support due to the reduced number of restriction-site-associated loci. In contrast to the nuclear population-level graphs, the individual level mitochondrial ML tree (Fig. 3 - f, Mitochondrial ML) provided a maternal perspective on these relationships. Despite a limited number of variants, the mitochondrial genome recovered distinct maternal lineages that aligned cleanly with geographic origin, clustering individuals into clear clades corresponding to Ethiopia, Yemen, Somaliland, and India. Overall, genome-wide and mitochondrial analyses consistently demonstrate robust genetic differentiation between the Indian laboratory colonies and invasive field populations.

### Admixture analysis of *An. stephensi* populations

**Fig. 4:**
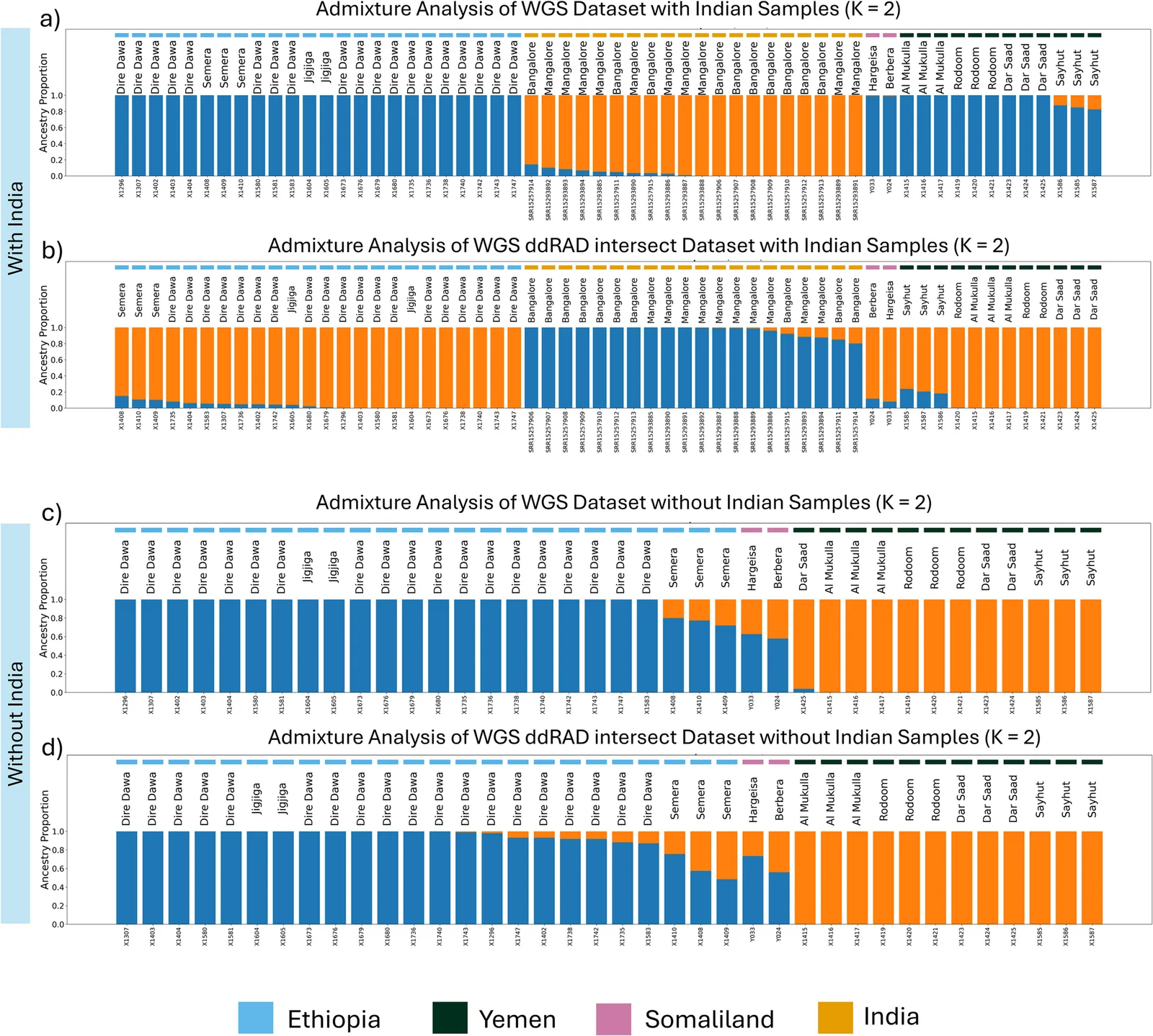
Admixture analyses plots of *An. stephensi* populations based on biallelic variants, WGS data (with India – a), without India – c)), WGS ddRAD intersection data (with India – b), without India – d)). The panels (**a**) and (**b**) represent an admixture plots with K = 2, where three genetic clusters were inferred based on five-fold cross-validation. The panels (**c**) and (**d**) shows an admixture analysis with K = 2, focusing only on the Ethiopia, Somaliland and Yemen populations

To explore genetic ancestry and population differentiation among *An. stephensi* populations, we performed ADMIXTURE analysis using multiple genomic datasets: whole-genome variant data, WGS ddRAD intersect data and ddRAD data (Fig. 4). The optimal number of genetic clusters (K) was determined based on five-fold cross-validation for each analysis (Supplementary Table 2).

Panels (a) and (b) in Fig. 4 illustrate ADMIXTURE plots at K = 2 using WGS data and the WGS ddRAD intersect data, respectively, including Indian samples. In both plots, Indian populations clearly separate from the Ethiopian, Yemen, and Somaliland groups, indicating strong genetic differentiation. It is important to notice that in both of these admixture plots, at K = 2, Sayhut samples show partial assignment to the same ancestry component as the Indian laboratory colony; however, this pattern should be interpreted cautiously, as clustering at low K values may not fully capture underlying population structure. Similarly, some Indian samples show partial assignment to ancestry components present in HoA and Yemen populations at low K valuesm. However, these patterns are not consistently observed at higher K values (Supplementary data file 3). In WGS ddRAD intersect admixture plot (panel b) all samples from Semera, Somaliland and a subset of the Dire Dawa samples also share anscestry with Indian laboratory reared colony samples.

Panels c) and d) depict ADMIXTURE analyses (K = 2) excluding Indian populations, using WGS (panel c) and WGS ddRAD intersect variants (panel d). These analyses reinforce the differentiation between Ethiopian and Yemen populations, with Ethiopian samples predominantly assigned to one ancestry component (blue) and Yemen samples to another (orange). Some admixed individuals, particularly from the Semera in Ethiopia and Somaliland, suggest limited ongoing gene flow or ancestral admixture.

Finally, ADMIXTURE results based solely on ddRAD data (see supplementary Fig. 1), with the optimal cluster number K = 8. This fine-scale analysis demonstrates substantial sub-structuring within and between populations, capturing detailed patterns of genetic ancestry and revealing high-resolution differentiation among Ethiopian, Yemen, and Somaliland populations. Multiple genetic clusters within each region indicate complex demographic histories.

Together, these ADMIXTURE results consistently demonstrate significant genetic differentiation between the Indian laboratory-reared colonies and field populations from Ethiopia, Yemen, and Somaliland. The analyses further highlight finer-scale structure within the HOA and the Yemen populations, suggesting varied historical connectivity, gene flow patterns, and localized adaptive evolution within this invasive malaria vector. Because ADMIXTURE results at low K values can reflect model constraints rather than true admixture, these patterns should be interpreted with caution.

### Detection of recent selective sweeps using H12 statistics

**Fig. 5:**
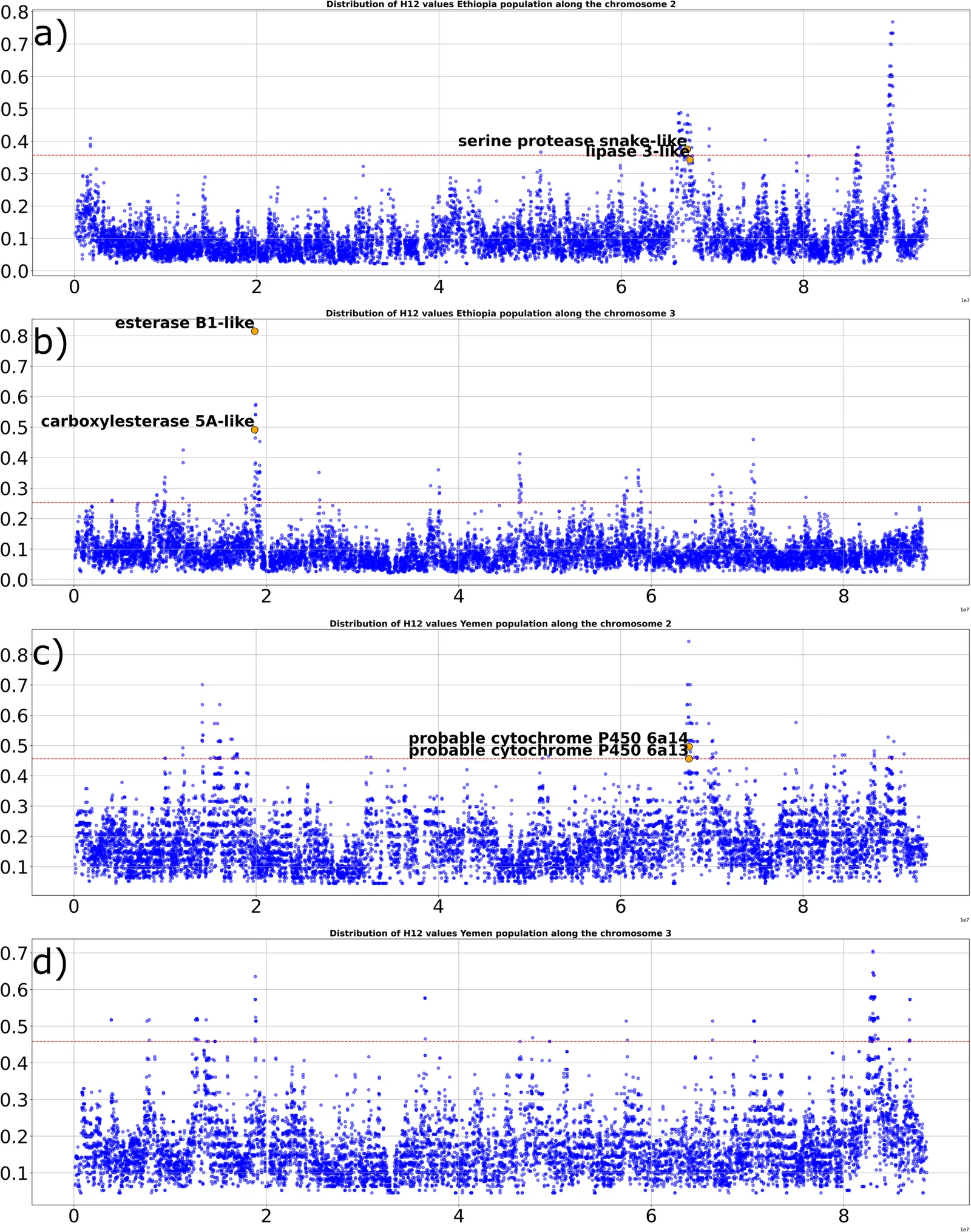
Chromosome wide distributions of H12 values in invasive *An. stephensi* populations. H12 values were calculated in 100 kb windows along autosomal chromosomes. The y-axis represents H12 values, a measure of haplotype homozygosity indicative of recent selective sweeps, while the x-axis shows genomic position (in base pairs). The red dashed line indicates the 99th percentile threshold used to identify candidate regions under selection. Panels (**a**) and (**b**) show H12 distributions for the Ethiopian population across chromosome 2 and chromosome 3, respectively. Panels (**c**) and (**d**) show H12 distributions for the Yemen population across chromosome 2 and chromosome 3, respectively. Genes labeled on the plots represent those located within regions exhibiting both elevated H12 values and elevated FST values (above the 99th percentile), highlighting candidate loci under selection

To identify regions of the genome under recent positive selection, we calculated the H12 statistic across Chromosomes 2 and 3 for *An. stephensi* populations from Ethiopia, Yemen (Fig. 5). H12 is a haplotype homozygosity-based statistic designed to detect recent selective sweeps by capturing high-frequency haplotype clusters indicative of positive selection.

In chromosome 2 both Ethiopian and Yemen populations show widespread and elevated H12 values. For Chromosome 3, a similar pattern is observed and shows elevated H12 values throughout the chromosome, with distinct peaks. The shared peaks between Ethiopian and Yemen populations suggest the possibility of selective pressures acting on common loci, which may be involved in traits relevant to survival in natural environments specific to HOA and Yemen.

Genes highlighted within candidate regions were identified following a comprehensive survey of functional annotations for all genes located in windows with H12 values exceeding the 99th percentile. Although several prominent peaks were detected across chromosomes, not all high H12 regions contained genes with known or clearly relevant functions associated with mosquito adaptation. The selective sweep signals in Ethiopian and Yemen populations suggest that natural selection is actively shaping the genetic architecture of *An. stephensi* in these regions, potentially in response to environmental challenges such as insecticides, climate variability, and pathogen interactions. Further investigation of the genes underlying these peaks that are also fixed between the two populations will provide insights into the adaptive mechanisms influencing mosquito populations in these regions. The sex chromosome was not considered for the interpretations.

Calculating Runs of homozygosity (RoH) alongside H12 is critical, as it helps distinguish selective sweeps driven by recent positive selection, which typically produce localized haplotype homozygosity from broader genomic regions of homozygosity caused by demographic events, such as inbreeding or population bottlenecks. We calculated RoH analysis along H12 calculation for the chromosomes of each individual within each population to investigate potential signals of inbreeding. The HOA and Yemen populations exhibited substantial amount of RoHs, a genomic signature characteristic of recently established invasive populations, consistent with previous observations [11]. However, the significant H12 signals identified in our populations are not within significant ROHs, and we ensured robustness in our selection analysis by considering only H12 peaks exceeding the 99th percentile threshold to infer selective sweeps (Supplementary data file 2).

### Sliding-window F_ST_ analysis of population differentiation

**Fig. 6:**
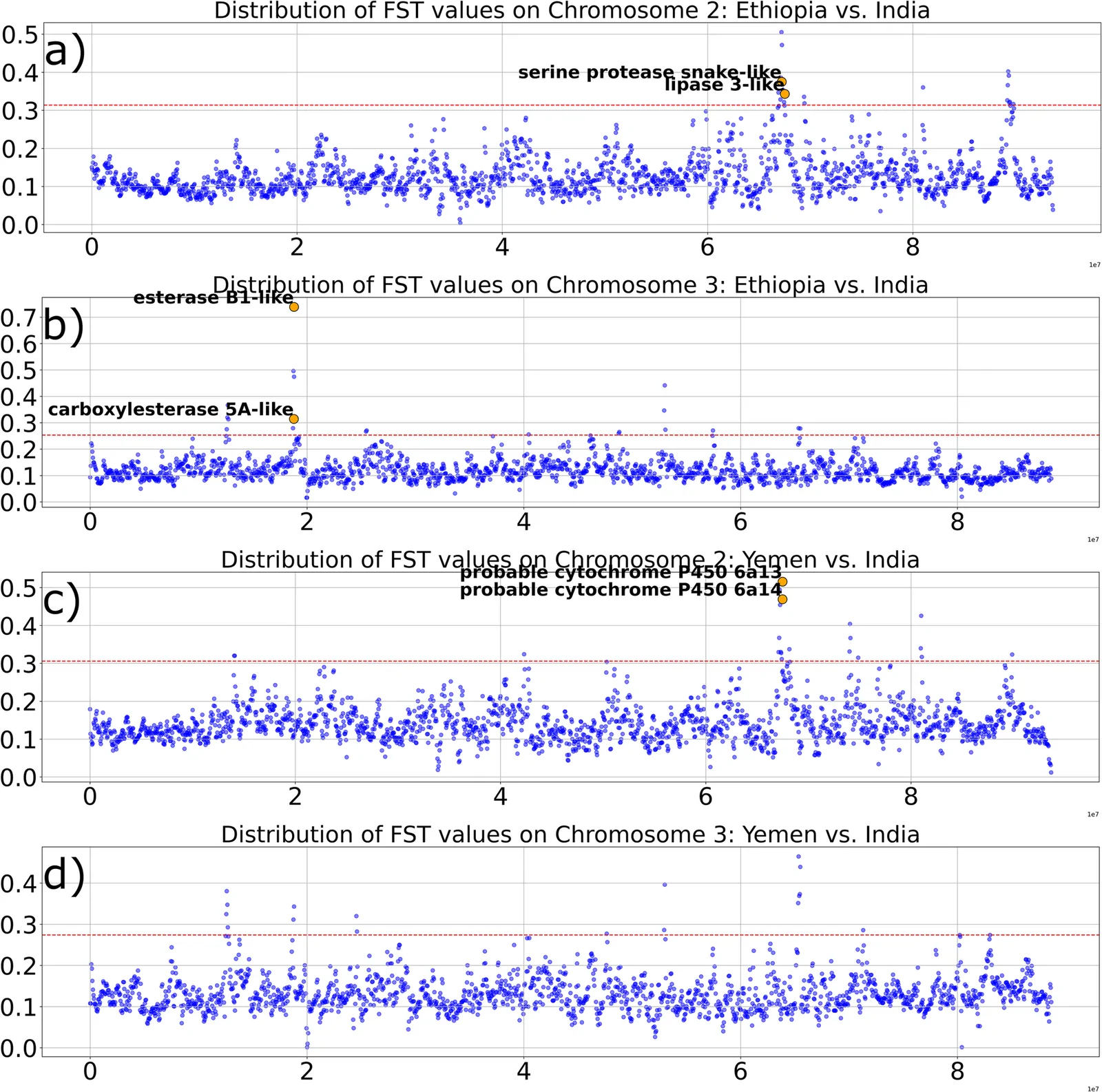
Genome-wide F_ST_ analysis of *An. stephensi* populations from Ethiopia and Yemen against India. Each scatter plot represents genome-wide pairwise F_ST_ values calculated between different population comparisons to assess genetic differentiation. The x-axis represents genomic position (in base pairs), while the y-axis shows F_ST_ values, with higher values indicating greater genetic divergence. The red dashed line indicates the 99th percentile threshold used to identify candidate regions with high differentiation. These results highlight the genetic distinctiveness of the Indian colony and reveal potential regions of local adaptation in Ethiopian and Yemeni *An. stephensi* populations. Genes labeled on the plots represent those located within regions showing both elevated H12 values and elevated F_ST_ values (above the 99th percentile)

To quantify genetic differentiation among *An. stephensi* populations from Ethiopia, Yemen, and a laboratory-reared Indian colony, we calculated pairwise F_ST_ values across the genome using a sliding-window approach (Fig. 6).

### Separate comparisons of Ethiopia vs. India & Yemen vs. India

Comparisons between Ethiopian and Indian populations, as well as Yemeni and Indian populations, reveal consistently high F_ST_ values across the genome, with multiple peaks of elevated differentiation. These results indicate strong genetic divergence between field-collected populations and the Indian laboratory colony, likely due to genetic drift and founder effects in the lab-reared population. The peaks in F_ST_ suggest regions of potential local adaptation in the Ethiopian and Yemen populations that have diverged from the Indian colony.

### Yemen and Ethiopia vs. India

When Ethiopian and Yemeni populations are combined and compared against the Indian colony, the genome-wide F_ST_ pattern remains similar, reinforcing the distinct genetic structure of the field populations compared to the Indian colony (see Supplementary Fig. 2). The high F_ST_ values suggest that the Indian colony has been genetically isolated from wild populations for an extended period, leading to substantial differentiation.

### Yemen vs. Ethiopia

The comparison between Yemeni and Ethiopian populations shows moderate F_ST_ values (compared to the F_ST_ values against Indian samples) across the genome. However, some genomic regions exhibit elevated F_ST_, suggesting that they may contain loci that are under differential selection or involved in local adaptation specific to one of the field populations. These peaks may indicate genetic variants associated with environmental factors such as insecticide resistance, climate adaptation, or host preference.

The F_ST_ results support the population structure observed in PCA and admixture analyses, confirming that Ethiopian and Yemeni populations are genetically distinct but more closely related to each other than to the Indian laboratory colony. The presence of highly differentiated genomic regions suggests the action of selective pressures unique to each population’s ecological and environmental conditions.

### Evaluation of ddRAD markers for detecting selection signals

To evaluate whether reduced-representation markers capture signals of adaptive evolution in *An. stephensi*, we compared patterns detected using ddRAD markers to those identified using WGS data. Because informative polymorphic sites between restriction enzyme cut sites can vary across the *An. stephensi* genome, the ability of ddRAD markers to detect selective sweeps may differ from expectations based on other systems. Supplementary Fig. 3 illustrates the distribution of H12 values across chromosomes 2 and 3, highlighting that while ddRAD data recaptured several peaks observed in WGS data, the correspondence was not consistent. In Ethiopian samples, some of the WGS and ddRAD H12 peaks aligned on chromosome 2, albeit with reduced magnitude in ddRAD, whereas chromosome 3 showed poor concordance, indicating a possible limitation of ddRAD in detecting certain selection signals. In Yemen, ddRAD captured a subset of strong WGS peaks on both chromosomes, suggesting partial overlap in detected selective sweeps.

Supplementary Fig. 4 demonstrates how population differentiation signals (F_ST_)between Ethiopia and Yemen compare between WGS and ddRAD variants. WGS-based F_ST_ profiles showed moderate genome-wide structure with several high-differentiation regions, while ddRAD-based F_ST_ values showed limited correspondence on chromosome 2 but revealed overlap with some of the WGS peaks on chromosome 3.

Consistent with previous findings, these results also suggest that ddRAD, may lack the resolution of WGS for fine-scale mapping, particularly in regions with complex or subtle signals [32].

### Nucleotide diversity across populations in comparison to H12 and F_ST_ peaks

Nucleotide diversity (π) was estimated along the autosomal chromosomes to evaluate patterns of genetic variation across *An. stephensi* populations and to find the most informative regions within invasive population in the context nucleotide variations. Further we wanted to check how nucleotide diversity in *An. stephensi* genomes would change with and without Indian lab-reared colony samples. In samples including Indian laboratory-reared colonies (Supplementary Fig. 5a and b), multiple regions showed consistent π values, with distinct peaks exceeding the 99th percentile. However, upon exclusion of Indian samples (Supplementary Fig. 5c and d), while the overall distribution of nucleotide diversity remained consistent, a subtle decrease in the magnitude and number of high-diversity regions was observed, suggesting the Indian lab population contributes slightly to diversity, likely due to its distinct but inbred (lab reared) demographic background.

Several genomic regions exhibiting low nucleotide diversity overlapped with high H12 and high F_ST_ values in Ethiopian and Yemeni populations. For instance, on chromosome 2, regions containing serine protease snake-like and lipase 3-like genes showed depressed π values (Supplementary Fig. 5, a and c), elevated H12 signals in Ethiopia (Fig. 5a), indicating a possible population-specific selective sweep. Similarly, probable cytochrome P450 6a14 and 6a13 on chromosome 2 showed a concordant pattern in Yemen, marked by reduced nucleotide diversity (Supplementary Figs. 5, b and d), supporting a scenario of local adaptation potentially driven by insecticide selection pressure. These overlapping signals collectively point to adaptive evolutionary events in distinct populations, where selection has driven alleles to high frequencies, reducing local genetic variation while increasing inter-population divergence.

## Discussion

Our analyses provide a comprehensive assessment of the genetic structure, differentiation, and adaptive evolution of *An. stephensi* populations from Ethiopia, Yemen, Somaliland and an Indian laboratory-reared colony. By integrating multiple genomic analyses, including PCA, phylogenetics, admixture analysis, haplotype-based selection scans, and F_ST_ differentiation measures, we reveal key insights into the population structure and evolutionary trajectories of this invasive malaria vector. Further, we compare the four genomic variant datasets in their capacity to characterize population structure in HOA and Yemen.

### All genomic datasets reveal population differentiation between Ethiopia, Somaliland, Yemen, and India

PCA and TreeMix population graphs across all data sets highlight genetic differentiation between the Indian lab reared colony and field-collected populations from Ethiopia and Yemen. The Indian laboratory-reared colony consistently forms a distinct cluster across all analyses, exhibiting a high degree of genetic drift in population graph models and indicating significant divergence from wild populations. This separation is likely driven by genetic drift, inbreeding, and potential laboratory adaptations, as seen in other captive mosquito colonies [20, 33]. In contrast, Ethiopian, Somaliland and Yemen populations are more closely related, clustering together in both PCA and TreeMix analyses. While they share substantial genetic similarity, subtle differentiation is evident.

Using multiple genomic datasets, we examined population structure and adaptive evolution in invasive *An. stephensi* populations. Each genomic dataset exhibited distinct capabilities in capturing genetic differentiation, highlighting their respective strengths and limitations. The autosomal variants from WGS data showed strong potential in revealing broad population structures, clearly distinguishing Ethiopian, Yemeni, and Indian laboratory reared colony samples along principal component axes. Notably, the WGS dataset provided resolution (while capturing differences within different parts of the genome), separating samples geographically and identifying nuances such as the distinct genetic composition of the Sayhut population in Yemen and connectivity among Semera samples with Yemen samples collected from sites close to Ethiopia.

In contrast, the ddRAD dataset, which involves fewer loci but higher sample throughput, successfully captured detailed intra-regional genetic structuring and was particularly effective at distinguishing between sub populations within the HOA and Yemen. The fine-scale resolution (geographical) provided by ddRAD was highlighted by ADMIXTURE analysis (optimal K = 8), clearly captured the complex substructure indicative of localized adaptations and demographic histories within these regions (Supplementary Fig. 1). However, there was a minor discrepancy between the magnitudes of cluster separation between HOA samples and Indian laboratory-reared colony samples when WGS data compared to WGS ddRAD intersect data in PCA (Fig. 2 panel c). This discrepancy may be partly due to biases in the subset of loci retained in the WGS ddRAD intersect dataset. In addition, the substantially lower number of markers in this dataset reduces the overall information content, making it more susceptible to stochastic variation and noise compared to the full WGS dataset. Additionally, while the higher geographic coverage of the ddRAD dataset successfully resolved a deep, well-supported split between a northern cluster (Yemen, Bati, and Semera) and a southern HOA cluster, it exhibited lower bootstrap support at shallower intra-regional nodes. This indicates that while reduced-representation data effectively captures broad macro-geographic barriers, it possesses reduced power to stabilize fine-scale regional branch topologies compared to genome-wide datasets (Fig. 3, ddRAD only).

Mitochondrial genome data, although providing valuable information regarding maternal lineage diversification and historical gene flow, showed significant limitations in population resolution compared to nuclear genomic datasets. The relatively low number of mitochondrial variants (62 variants) resulted in substantial overlap among samples in PCA analyses, reducing clarity in identifying distinct population clusters. However, unlike the nuclear datasets which represent recombining population level networks, the haploid mitochondrial data were highly effective in individual level ML phylogenetic tree analyses. The maternal tree clearly resolved distinct geographic clades, complementing the TreeMix graphs by capturing deeper maternal lineage divergences, particularly notable among Yemeni populations.

These findings provide valuable guidance for future researchers in selecting the most appropriate genomic data type, WGS, ddRAD, or mitochondrial based on the specific goals of their study, as well as considerations such as budget constraints, technical expertise, and available computational resources during the experimental design phase. Additionally, our analyses revealed that the Sayhut samples from Yemen are genetically distinct from other Yemen populations. Increasing the sample size at these geographically dispersed locations and comparing them with *An. stephensi* populations from neighboring countries, as we intend in our subsequent study, will be crucial to further elucidate the genetic origins and potential introduction routes of *An. stephensi* in this region. Because ddRAD markers target genomic regions adjacent to restriction enzyme cut sites, the distribution of informative polymorphisms across the *An. stephensi* genome may influence the ability of reduced representation data to capture signals of selection. Our results suggest that ddRAD markers capture major population structure patterns but does not fully detect the selective sweeps identified using WGS.

### Analysis of genomic differentiation reveals geography-specific signals of selection in invasive range

Genome-wide F_ST_ analyses of whole genome sequences confirm significant genetic differentiation between the Indian laboratory-reared colony and the field populations, with consistently high differentiation across the genome. The Ethiopian and Yemen populations, while genetically closer, also show regions of elevated F_ST_, indicative of differential selection or local adaptation (Supplementary Fig. 2). These differentiated regions may contain genes associated with vector competence, insecticide resistance, or other ecologically relevant traits. Notably, the highest F_ST_ peaks coincide with some of the strongest selection signals detected in the H12 analysis, further supporting the role of adaptive evolution in shaping genomic divergence.

Haplotype homozygosity-based selection scans using the H12 statistic reveal multiple regions under strong selection in Ethiopian and Yemen populations, particularly on chromosomes 2 and 3. These selective sweeps are reinforcing the idea that field populations are undergoing continuous evolutionary pressures, likely from insecticide exposure, climatic adaptation, or host preference shifts [17]. The shared peaks between Ethiopian and Yemen populations suggest selection on common adaptive loci, potentially reflecting convergent evolution in response to similar environmental challenges [11].

Comparison between WGS and ddRAD datasets revealed limited concordance in detecting signals of selection and fixation, with ddRAD capturing only a subset of peaks identified by WGS (Supplementary Figs. 3 and 4). This highlights the reduced resolution of ddRAD for detecting fine-scale selective sweeps and reinforces the utility of WGS for precise localization of adaptive loci.

### Ethiopia-specific signals of selection

We identified four genes potentially associated with adaptive characteristics, within the genomic regions with signals of selective sweeps specific to Ethiopia (genomic regions with Garud’s H12 values above 99th percentile). All four of them were fixed (windows with F_ST_ values above 99th percentile) between Ethiopian population and Indian lab colony. Only *Carboxylesterase 5 A-Like* was the gene fixed between Ethiopian and Yemen populations as well as with a signal of selective sweep in Ethiopian population.

*Carboxylesterase 5 A-Like* and *Esterase B1-Like* genes, located on the third chromosome had signals of selection and are important for the insecticide resistance in Ethiopia. Carboxylesterases are enzymes that hydrolyze carboxylic esters and are involved in detoxification processes [34]. In insects, they play roles in metabolizing xenobiotics, including insecticides. Certain carboxylesterases showed enhanced hydrolytic activity toward permethrin, a pyrethroid insecticide. Cells expressing these enzymes demonstrated increased tolerance to permethrin, underscoring their role in detoxification and resistance mechanisms [35–38]. In *Culex pipiens quinquefasciatus*, resistance to organophosphorus (OP) insecticides has been linked to the amplification of the esterase B1 gene, resulting in increased production of the esterase enzyme. This overproduction enhances the mosquito’s ability to detoxify insecticides, leading to higher resistance levels. The overproduced esterases, such as B1, metabolize and sequester insecticides, reducing their toxicity. This mechanism is a common adaptation in mosquitoes exposed to chemical control measures [39–42].

The two Chromosome 2 genes located within the regions with signals of high fixation and selection, *serine protease snake-like* and *lipase 3-like*, are important in organisms such as *An. gambiae* and *Drosophila* for immune responses and survival under challenging conditions respectively. In *Anopheles gambiae*, certain CLIP-domain serine proteases (CLIPs) are essential for the melanization immune response, a defense mechanism where pathogens are encapsulated in melanin to restrict their spread [43]. For instance, CLIPB10 has been identified as a terminal protease in the regulatory network controlling melanization, directly activating prophenoloxidase (proPO), the enzyme responsible for melanin synthesis [44]. In *Drosophila*, Lipase 3 is significantly upregulated during prolonged nutrient deprivation, suggesting its involvement in mobilizing lipid reserves to provide energy under starvation conditions. This adaptation is vital for survival during periods of food scarcity [45].

### Yemen-specific signals of selection

Our analyses of Yemen-specific selection revealed two genes on chromosome 2 that lie within regions exhibiting strong signals of selective sweeps in the Yemen population (Garud’s H12 > 99th percentile) that are also highly differentiated (F_ST_ > 99th percentile) from the Indian lab colony. Both of these genes, *Probable Cytochrome P450 6a13* and *6a14*, are important for insecticide resistance. Cytochrome P450 enzymes are involved in the metabolism of endogenous and exogenous compounds. In insects, certain P450s have been associated with insecticide resistance by metabolizing toxic compounds. *Probable Cytochrome P450 6a13* and *6a14* (In *Culex quinquefasciatus*) are likely involved in the metabolism of various substrates, including potential roles in insecticide resistance. Functional studies are needed to determine their specific roles in *Anopheles stephensi* [46, 47].

The selection signals identified near specific genes provide insights into the potential drivers of adaptation shaping *An. stephensi* populations in the Horn of Africa and Yemen, such as insecticide resistance, environmental pressures, or pathogen interactions. These findings have important implications for guiding future research aimed at characterizing vulnerable genomic loci, as well as informing vector control strategies and policy development. However, it is important to note that gene annotation in the *An. stephensi* genome remains incomplete and less robust compared to model organisms such as humans or *Drosophila melanogaster*. Therefore, further functional validation is essential to confirm the roles of the candidate genes identified in this study.

Different signals of selection between Ethiopia and Yemen may be due to the distinct environmental and anthropogenic pressures acting on *An. stephensi* populations in these regions [48]. These divergent adaptive trajectories have important implications for control strategies. Locally adapted mosquito populations may exhibit variable resistance profiles, reducing the efficacy of uniform interventions. This underscores the need for regionally tailored vector control programs informed by population genomic data [49]. We did not observe signals of selection at the *kdr* mutation site within the voltage-gated sodium channel (*VGSC*) gene in *Anopheles stephensi*, corroborating previous findings that suggest limited relevance of *kdr*-mediated resistance in this species in the invasive region [50].

## Conclusion

In conclusion, Whole-genome sequencing provided the most comprehensive view of genomic differentiation and adaptive signals across invasive *An. stephensi* populations. With a higher number of samples, ddRAD markers captured major population structure patterns but was not capable of comprehensively identify the selective signals identified with WGS, suggesting that reduced representation data may be useful for large-scale surveillance but may have limitations for identifying fine scale adaptive loci.

Based on these findings we can suggest that the two main selective forces in Ethiopian and Yemeni population could be insecticides. In Ethiopia selective forces exerted by pathogens or microbes could be another factor that is driving the evolution. Further, these findings indicate that vector control considerations should be made carefully because the selection pressure of insecticide use without consideration for resistance management may exacerbate and drive issues of invasion.

This study establishes a foundation for future investigations into the fine-scale population structure of *An. stephensi* within the Horn of Africa and Yemen, as well as for functional studies aimed at elucidating the adaptive mechanisms facilitating the species’ establishment and persistence in these regions.

## Supplementary Information

Supplementary Material 1.

Supplementary Material 2.

Supplementary Material 3.

Supplementary Material 4.

Supplementary Material 5.

## Data Availability

All whole-genome and a portion of the ddRAD sequencing data generated in this study have been deposited in the NCBI Sequence Read Archive (SRA) under BioProject PRJNA1359442 (reviewers link - https://dataview.ncbi.nlm.nih.gov/object/PRJNA1359442?reviewer=d8ldtmj5tofsmq7ghi7tcq279o), together with associated metadata. Additional ddRAD datasets previously published under a separate study are available in the SRA under Bioproject PRJNA888109 (10). The laboratory-reared Indian reference colony samples are available under BioProject PRJNA746765 (18). More information on all samples (locations) are provided in the supplementary file “Supplementary-data_Sample_Details.xlsx.” The custom shell and Python scripts used for data processing and analysis are available at the GitHub repository: ( [https://github.com/IGcodes/HOAnYemen_Anstep-ga-repo](https:/github.com/IGcodes/HOAnYemen_Anstep-ga-repo) ).

## References

[1] World Health Organization. World malaria report 2024: addressing inequity in the global malaria response. World Health Organization. 2024. https://www.who.int/publications/i/item/9789240104440.

[2] Faulde MK, Rueda LM, Khaireh BA. First record of the Asian malaria vector Anopheles stephensi and its possible role in the resurgence of malaria in Djibouti, Horn of Africa. Acta Trop. 2014;139:39–43. 10.1016/j.actatropica.2014.06.016 25004439

[3] Carter TE, Yared S, Gebresilassie A, Bonnell V, Damodaran L, Lopez K, et al. First detection of Anopheles stephensi Liston, 1901 (Diptera: culicidae) in Ethiopia using molecular and morphological approaches. Acta Trop. 2018;188:180–6. 10.1016/j.actatropica.2018.09.001 30189199

[4] Ali S, Samake JN, Spear J, Carter TE. Morphological identification and genetic characterization of Anopheles stephensi in Somaliland. Parasit Vectors. 2022;15(1):247. 10.1186/s13071-022-05339-y PMC9270831 35804441

[5] Subbarao SK, Nanda N, Rahi M, Raghavendra K. Biology and bionomics of malaria vectors in India: existing information and what more needs to be known for strategizing elimination of malaria. Malar J. 2019;18(1):396. 10.1186/s12936-019-3011-8 PMC6892149 31796010

[6] Thomas S, Ravishankaran S, Justin NA, Asokan A, Mathai MT, Valecha N, et al. Resting and feeding preferences of Anopheles stephensi in an urban setting, perennial for malaria. Malar J. 2017;16(1):111. 10.1186/s12936-017-1764-5 PMC5346253 28283033

[7] Assada M, Al-Hadi M, Esmail MA, Al-Jurban J, Alkawri A, Shamsan A, et al. Molecular Confirmation of Anopheles stephensi Mosquitoes in the Al Hudaydah Governorate, Yemen, 2021 and 2022. Emerg Infect Dis. 2024;30(7):1467–71. 10.3201/eid3007.240331 PMC11210655 38916721

[8] Al-Eryani SM, Irish SR, Carter TE, Lenhart A, Aljasari A, Montoya LF, et al. Public health impact of the spread of Anopheles stephensi in the WHO Eastern Mediterranean Region countries in Horn of Africa and Yemen: need for integrated vector surveillance and control. Malar J. 2023;22(1):187. 10.1186/s12936-023-04545-y PMC10278259 37337209

[9] Baheshm YA, Zayed A, Awash AA, Follis M, Terreri P, Samake JN, et al. Sequencing confirms Anopheles stephensi distribution across southern Yemen. Parasit Vectors. 2024;17(1):507. 10.1186/s13071-024-06601-1 PMC11657292 39696448

[10] Samake JN, Lavretsky P, Gunarathna I, Follis M, Brown JI, Ali S et al. Population genomic analyses reveal population structure and major hubs of invasive Anopheles stephensi in the Horn of Africa. Mol Ecol. 2023;32(21):5695-5708. 10.1111/mec.17136. PMC12822364 37795951

[11] Dennis TPW, Sulieman JE, Abdin M, Ashine T, Asmamaw Y, Eyasu A et al. The origin, invasion history and resistance architecture of Anopheles stephensi in Africa. bioRxiv. 2025 Mar 26:2025.03.24.644828.

[12] Carter TE, Yared S, Getachew D, Spear J, Choi SH, Samake JN, et al. Genetic diversity of Anopheles stephensi in Ethiopia provides insight into patterns of spread. Parasit Vectors. 2021;14(1):602. 10.1186/s13071-021-05097-3 PMC8665610 34895319

[13] Lavretsky P. Population Genomics Provides Key Insights into Admixture, Speciation, and Evolution of Closely Related Ducks of the Mallard Complex. In: Hohenlohe PA, Rajora OP, editors. Population Genomics: Wildlife. Cham: Springer International Publishing; 2021. pp. 295–330.

[14] Lajmi A, Glinka F, Privman E. Optimizing ddRAD sequencing for population genomic studies with ddgRADer. Mol Ecol Resour. 2025;25(5):e13870. 10.1111/1755-0998.13870 PMC12142716 37732396

[15] Oshaghi MA. mtDNA inheritance in the mosquitoes of Anopheles stephensi. Mitochondrion. 2005;5(4):266–71. 10.1016/j.mito.2005.05.001 16050989

[16] Lozada-Chavez AN, Lozada-Chavez I, Alfano N, Palatini U, Sogliani D, Elfekih S, et al. Adaptive genomic signatures of globally invasive populations of the yellow fever mosquito Aedes aegypti. Nat Ecol Evol. 2025;9(4):652–71. 10.1038/s41559-025-02643-5 PMC11976285 40155778

[17] Acford-Palmer H, Tadesse FG, Manko E, Phelan JE, Higgins M, Osborne A, et al. Genome wide population genetics and molecular surveillance of insecticide resistance in Anopheles stephensi mosquitoes from Awash Sebat Kilo in Ethiopia. Sci Rep. 2025;15(1):16443. 10.1038/s41598-025-95814-0 PMC12069653 40355632

[18] Samake JN, Yared S, Hassen MA, Zohdy S, Carter TE. Insecticide resistance and population structure of the invasive malaria vector, Anopheles stephensi, from Fiq, Ethiopia. Sci Rep. 2024;14(1):27516. 10.1038/s41598-024-78072-4 PMC11554808 39528579

[19] Zhou G, Taffese HS, Zhong D, Wang X, Lee MC, Degefa T et al. Resurgence of Clinical Malaria in Ethiopia and Its Link to Anopheles stephensi Invasion. Pathogens. 2024;13(9):748. 10.3390/pathogens13090748 PMC11435327 39338938

[20] Thakare A, Ghosh C, Alalamath T, Kumar N, Narang H, Whadgar S, et al. The genome trilogy of Anopheles stephensi, an urban malaria vector, reveals structure of a locus associated with adaptation to environmental heterogeneity. Sci Rep. 2022;12(1):3610. 10.1038/s41598-022-07462-3 PMC8897464 35246568

[21] Andrews S. FastQC: A Quality Control Tool for High Throughput Sequence Data. Babraham Bioinformatics. 2010. http://www.bioinformatics.babraham.ac.uk/projects/fastqc.

[22] Li H, Durbin R. Fast and accurate long-read alignment with Burrows-Wheeler transform. Bioinformatics. 2010;26(5):589–95. 10.1093/bioinformatics/btp698 PMC2828108 20080505

[23] Bolger AM, Lohse M, Usadel B. Trimmomatic: a flexible trimmer for Illumina sequence data. Bioinformatics. 2014;30(15):2114–20. 10.1093/bioinformatics/btu170 PMC4103590 24695404

[24] Van der Auwera GA, Carneiro MO, Hartl C, Poplin R, Del Angel G, Levy-Moonshine A, et al. From FastQ data to high confidence variant calls: the Genome Analysis Toolkit best practices pipeline. Curr Protoc Bioinf. 2013;43(1110):11. 0 1 – 0 33. 10.1002/0471250953.bi1110s43 PMC4243306 25431634

[25] Purcell S, Neale B, Todd-Brown K, Thomas L, Ferreira MA, Bender D, et al. PLINK: a tool set for whole-genome association and population-based linkage analyses. Am J Hum Genet. 2007;81(3):559–75. 10.1086/519795 PMC1950838 17701901

[26] Ueki M, Cordell HJ. Improved statistics for genome-wide interaction analysis. PLoS Genet. 2012;8(4):e1002625. 10.1371/journal.pgen.1002625 PMC3320596 22496670

[27] Pickrell JK, Pritchard JK. Inference of population splits and mixtures from genome-wide allele frequency data. PLoS Genet. 2012;8(11):e1002967. 10.1371/journal.pgen.1002967 PMC3499260 23166502

[28] Alexander DH, Novembre J, Lange K. Fast model-based estimation of ancestry in unrelated individuals. Genome Res. 2009;19(9):1655–64. 10.1101/gr.094052.109 PMC2752134 19648217

[29] Garud NR, Messer PW, Petrov DA. Detection of hard and soft selective sweeps from Drosophila melanogaster population genomic data. PLoS Genet. 2021;17(2):e1009373. 10.1371/journal.pgen.1009373 PMC7946363 33635910

[30] Browning BL, Tian X, Zhou Y, Browning SR. Fast two-stage phasing of large-scale sequence data. Am J Hum Genet. 2021;108(10):1880–90. 10.1016/j.ajhg.2021.08.005 PMC8551421 34478634

[31] Weir BS, Cockerham CC. Estimating F-Statistics for the Analysis of Population Structure. Evolution. 1984;38(6):1358–70. 10.1111/j.1558-5646.1984.tb05657.x 28563791

[32] Doublet M, Degalez F, Lagarrigue S, Lagoutte L, Gueret E, Allais S, et al. Variant calling and genotyping accuracy of ddRAD-seq: Comparison with 20X WGS in layers. PLoS ONE. 2024;19(7):e0298565. 10.1371/journal.pone.0298565 PMC11280156 39058708

[33] Chakraborty M, Ramaiah A, Adolfi A, Halas P, Kaduskar B, Ngo LT, et al. Hidden genomic features of an invasive malaria vector, Anopheles stephensi, revealed by a chromosome-level genome assembly. BMC Biol. 2021;19(1):28. 10.1186/s12915-021-00963-z PMC7876825 33568145

[34] Jackson C, Oakeshott JG, Sanchez-Hernandez J, Wheelock C. Carboxylesterases in the metabolism and toxicity of pesticides. Anticholinesterase Pesticides: Metabolism, Neurotoxicity and Epidemiology. Hoboken: John Wily & Sons; 2010. pp. 57–76.

[35] Feng X, Liu N. Functional Analyses of House Fly Carboxylesterases Involved in Insecticide Resistance. Front Physiol. 2020;11:595009. 10.3389/fphys.2020.595009 PMC7596742 33178052

[36] Gong Y, Li M, Li T, Liu N. Molecular and functional characterization of three novel carboxylesterases in the detoxification of permethrin in the mosquito, Culex quinquefasciatus. Insect Sci. 2022;29(1):199–214. 10.1111/1744-7917.12927 34048147

[37] Cattel J, Haberkorn C, Laporte F, Gaude T, Cumer T, Renaud J, et al. A genomic amplification affecting a carboxylesterase gene cluster confers organophosphate resistance in the mosquito Aedes aegypti: From genomic characterization to high-throughput field detection. Evol Appl. 2021;14(4):1009–22. 10.1111/eva.13177 PMC8061265 33897817

[38] Poupardin R, Srisukontarat W, Yunta C, Ranson H. Identification of carboxylesterase genes implicated in temephos resistance in the dengue vector Aedes aegypti. PLoS Negl Trop Dis. 2014;8(3):e2743. 10.1371/journal.pntd.0002743 PMC3961196 24651719

[39] Mouches C, Pasteur N, Berge JB, Hyrien O, Raymond M, de Saint Vincent BR, et al. Amplification of an esterase gene is responsible for insecticide resistance in a California Culex mosquito. Science. 1986;233(4765):778–80. 10.1126/science.3755546 3755546

[40] Mouches C, Pauplin Y, Agarwal M, Lemieux L, Herzog M, Abadon M, et al. Characterization of amplification core and esterase B1 gene responsible for insecticide resistance in Culex. Proc Natl Acad Sci U S A. 1990;87(7):2574–8. 10.1073/pnas.87.7.2574 PMC53732 2320576

[41] Qiao CL, Raymond M. The same esterase B1 haplotype is amplified in insecticide-resistant mosquitoes of the Culex pipiens complex from the Americas and China. Heredity (Edinb). 1995;74(Pt 4):339–45. 10.1038/hdy.1995.51 7538988

[42] Pasteur N, Nance E, Bons N. Tissue localization of overproduced esterases in the mosquito Culex pipiens (Diptera: Culicidae). J Med Entomol. 2001;38(6):791–801. 10.1603/0022-2585-38.6.791 11761376

[43] Cao X, Gulati M, Jiang H. Serine protease-related proteins in the malaria mosquito, Anopheles gambiae. Insect Biochem Mol Biol. 2017;88:48–62. 10.1016/j.ibmb.2017.07.008 PMC5586530 28780069

[44] Zhang X, Li M, El Moussawi L, Saab S, Zhang S, Osta MA, et al. CLIPB10 is a Terminal Protease in the Regulatory Network That Controls Melanization in the African Malaria Mosquito Anopheles gambiae. Front Cell Infect Microbiol. 2020;10:585986. 10.3389/fcimb.2020.585986 PMC7843523 33520733

[45] Hanschke L, Heier C, Maya Palacios SJ, Ozek HE, Thiele C, Bauer R, et al. Drosophila Lipase 3 Mediates the Metabolic Response to Starvation and Aging. Front Aging. 2022;3:800153. 10.3389/fragi.2022.800153 PMC9261307 35821816

[46] Gong Y, Li T, Li Q, Liu S, Liu N. The Central Role of Multiple P450 Genes and Their Co-factor CPR in the Development of Permethrin Resistance in the Mosquito Culex quinquefasciatus. Front Physiol. 2021;12:802584. 10.3389/fphys.2021.802584 PMC8792746 35095564

[47] Chandor-Proust A, Bibby J, Regent-Kloeckner M, Roux J, Guittard-Crilat E, Poupardin R, et al. The central role of mosquito cytochrome P450 CYP6Zs in insecticide detoxification revealed by functional expression and structural modelling. Biochem J. 2013;455(1):75–85. 10.1042/BJ20130577 PMC3778711 23844938

[48] Hancock PA, Hendriks CJM, Tangena JA, Gibson H, Hemingway J, Coleman M, et al. Mapping trends in insecticide resistance phenotypes in African malaria vectors. PLoS Biol. 2020;18(6):e3000633. 10.1371/journal.pbio.3000633 PMC7316233 32584814

[49] Moyes CL, Athinya DK, Seethaler T, Battle KE, Sinka M, Hadi MP, et al. Evaluating insecticide resistance across African districts to aid malaria control decisions. Proc Natl Acad Sci U S A. 2020;117(36):22042–50. 10.1073/pnas.2006781117 PMC7486715 32843339

[50] Samake JN, Yared S, Getachew D, Mumba P, Dengela D, Yohannes G, et al. Detection and population genetic analysis of kdr L1014F variant in eastern Ethiopian Anopheles stephensi. Infect Genet Evol. 2022;99:105235. 10.1016/j.meegid.2022.105235 PMC11959535 35123054

[51] Djadid ND, Gholizadeh S, Aghajari M, Zehi AH, Raeisi A, Zakeri S. Genetic analysis of rDNA-ITS2 and RAPD loci in field populations of the malaria vector, Anopheles stephensi (Diptera: Culicidae): implications for the control program in Iran. Acta Trop. 2006;97(1):65–74. 10.1016/j.actatropica.2005.08.003 16188214

[52] Hao YJ, Zou YL, Ding YR, Xu WY, Yan ZT, Li XD, et al. Complete mitochondrial genomes of Anopheles stephensi and An. dirus and comparative evolutionary mitochondriomics of 50 mosquitoes. Sci Rep. 2017;7(1):7666. 10.1038/s41598-017-07977-0 PMC5550476 28794438

[53] Samake JN, Lavretsky P, Gunarathna I, Follis M, Brown JI, Ali S, et al. Population genomic analyses reveal population structure and major hubs of invasive Anopheles stephensi in the Horn of Africa. Mol Ecol. 2023;32(21):5695–708. 10.1111/mec.17136 PMC12822364 37795951

[54] Li H. A statistical framework for SNP calling, mutation discovery, association mapping and population genetical parameter estimation from sequencing data. Bioinformatics. 2011;27(21):2987–93. 10.1093/bioinformatics/btr509 PMC3198575 21903627

[55] Chenna R, Sugawara H, Koike T, Lopez R, Gibson TJ, Higgins DG, et al. Multiple sequence alignment with the Clustal series of programs. Nucleic Acids Res. 2003;31(13):3497–500. 10.1093/nar/gkg500 PMC168907 12824352

[56] Edgar RC. MUSCLE: multiple sequence alignment with high accuracy and high throughput. Nucleic Acids Res. 2004;32(5):1792–7. 10.1093/nar/gkh340 PMC390337 15034147

