# Supplementary material for: Multiple genomic approaches reveal geographic structure and local selection signals in invasive Anopheles stephensi from the Horn of Africa and Yemen
Source: BMC Genomics. 2026 Jul 7;27:802. doi: 10.1186/s12864-026-13079-x (PMC13625371; doi:10.1186/s12864-026-13079-x)
Supplement: Supplementary file 1 [file 12864_2026_13079_MOESM1_ESM.pdf]

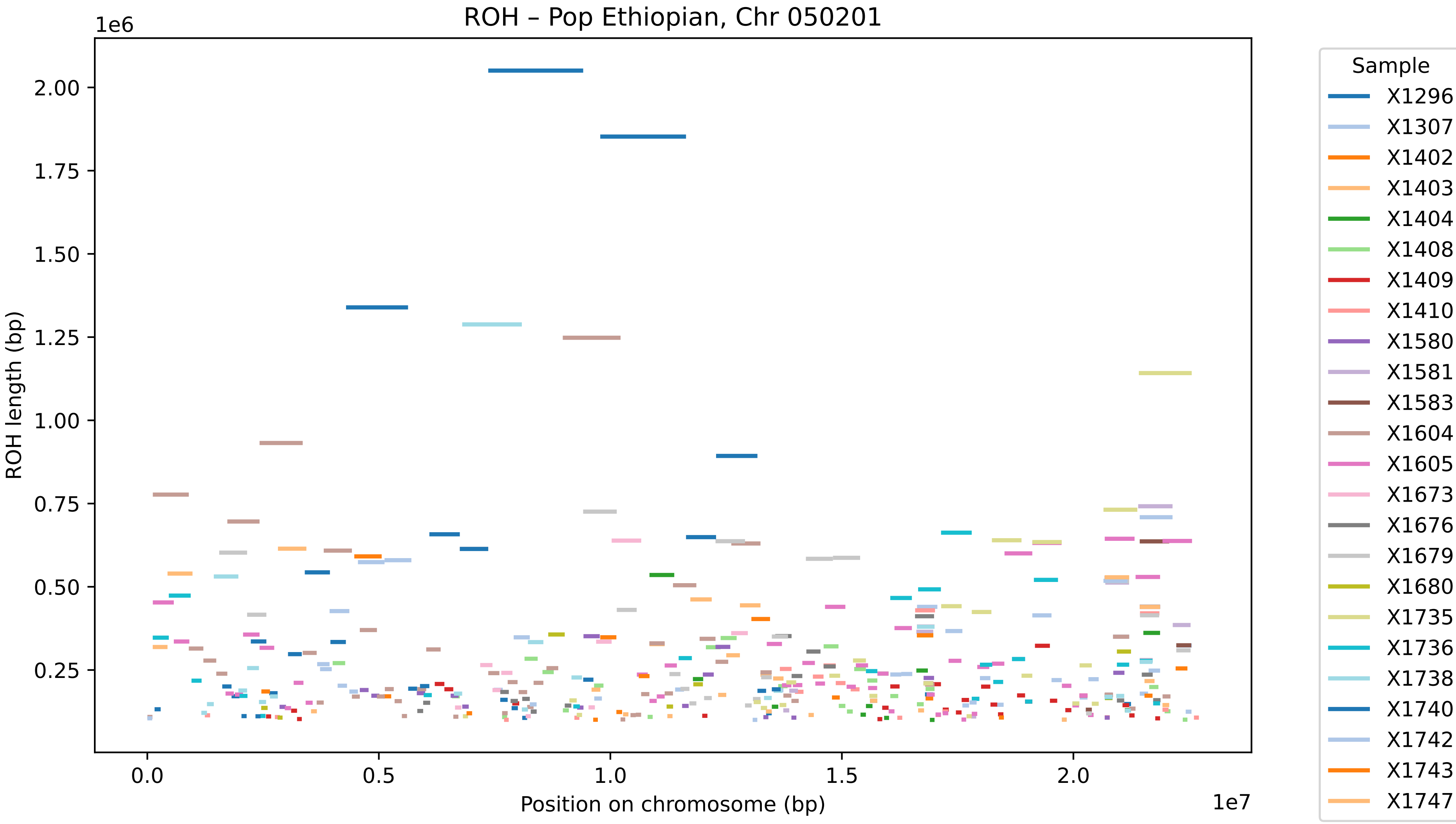

ROH – Pop Ethiopian, Chr 050201
1e6
2.00
1.75
1.50
1.25
1.00
0.75
0.50
0.25
ROH length (bp)
0.0
0.5
1.0
1.5
2.0
1e7
Position on chromosome (bp)
Sample
X1296
X1307
X1402
X1403
X1404
X1408
X1409
X1410
X1580
X1581
X1583
X1604
X1605
X1673
X1676
X1679
X1680
X1735
X1736
X1738
X1740
X1742
X1743
X1747

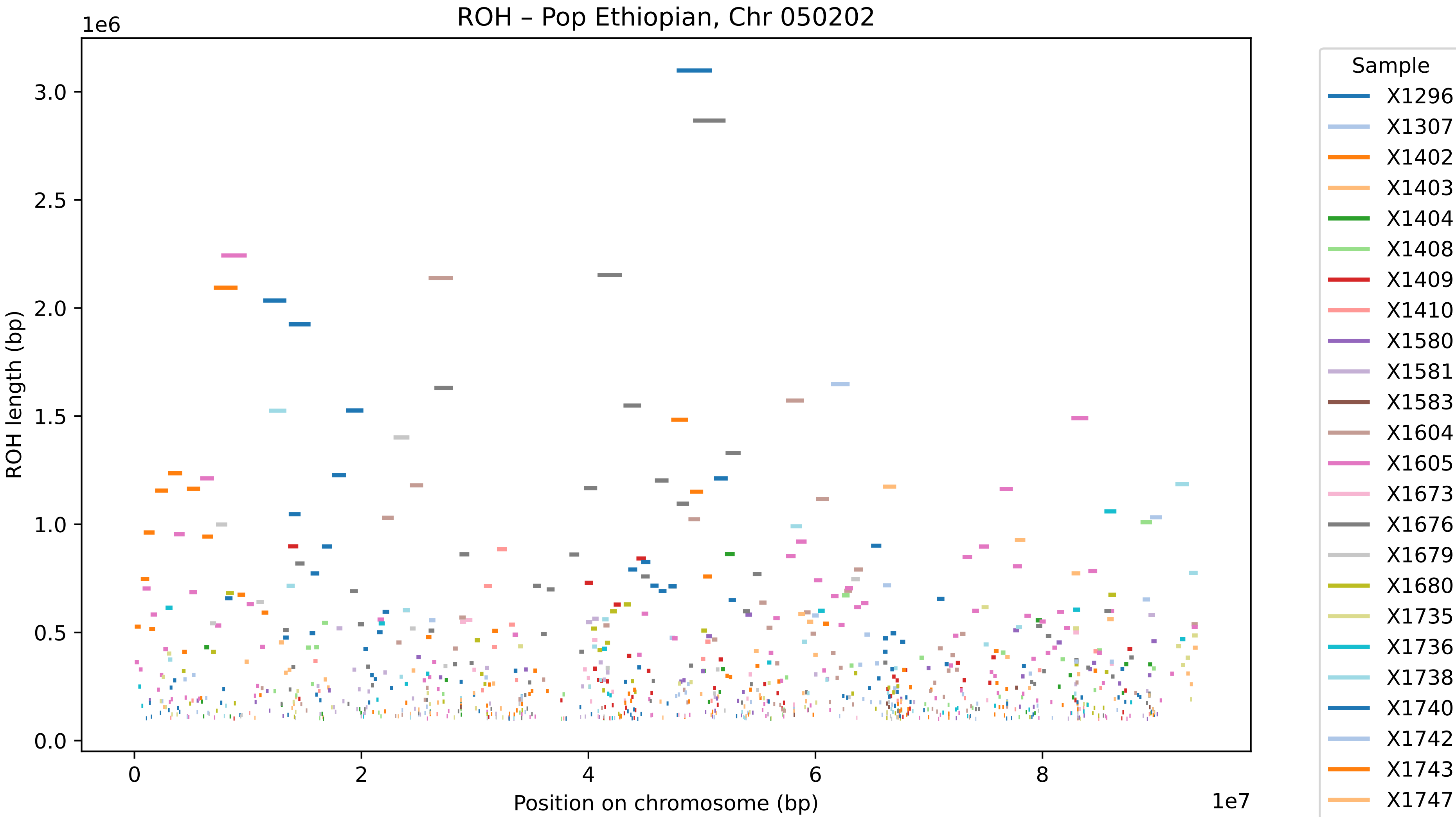

ROH – Pop Ethiopian, Chr 050202
1e6
3.0
2.5
2.0
1.5
1.0
0.5
0.0
ROH length (bp)
0
2
4
6
8
1e7
Position on chromosome (bp)
Sample
X1296
X1307
X1402
X1403
X1404
X1408
X1409
X1410
X1580
X1581
X1583
X1604
X1605
X1673
X1676
X1679
X1680
X1735
X1736
X1738
X1740
X1742
X1743
X1747

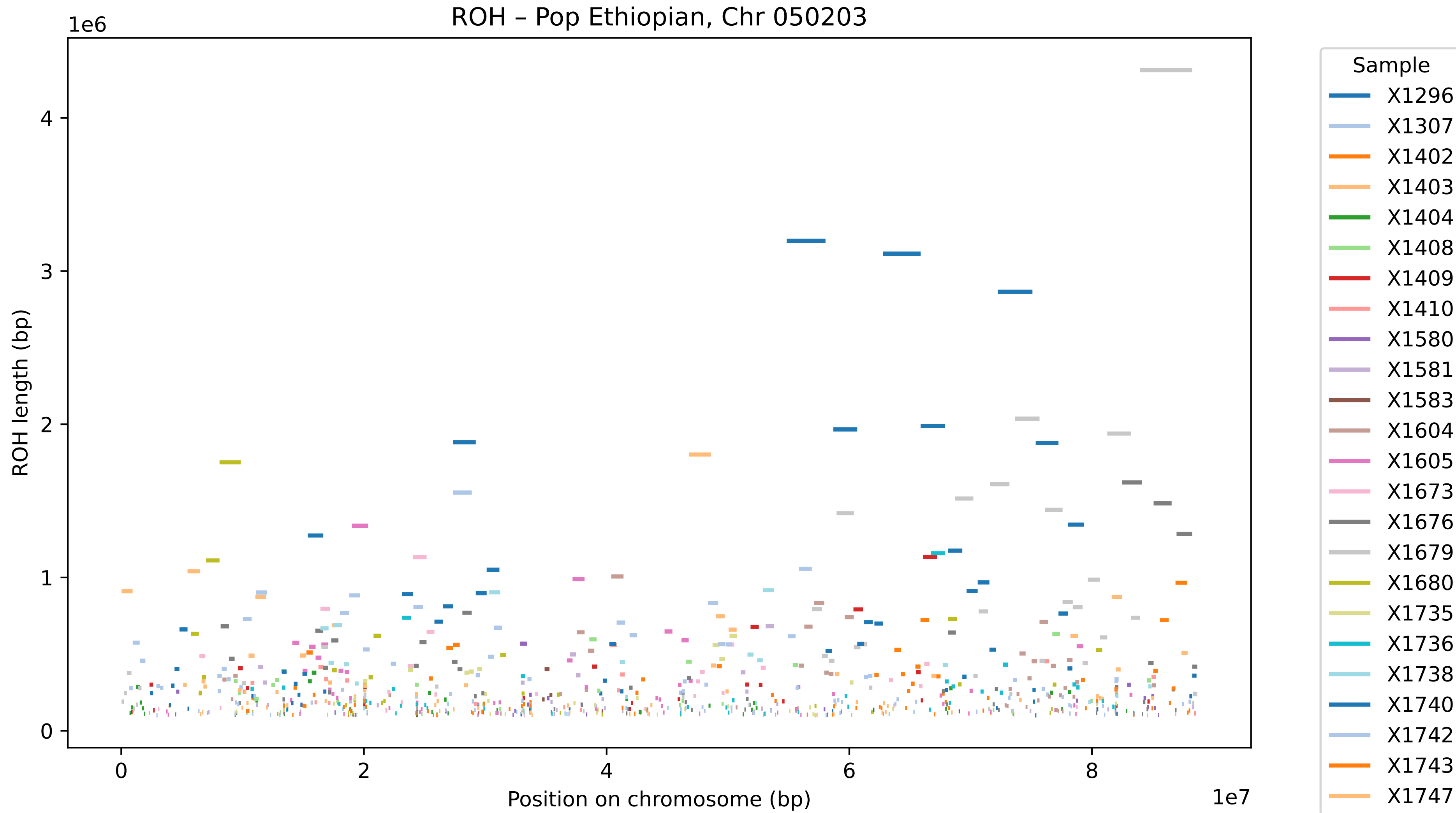

ROH – Pop Ethiopian, Chr 050203
1e6
4
3
2
1
0
ROH length (bp)
0
2
4
6
8
1e7
Position on chromosome (bp)
Sample
X1296
X1307
X1402
X1403
X1404
X1408
X1409
X1410
X1580
X1581
X1583
X1604
X1605
X1673
X1676
X1679
X1680
X1735
X1736
X1738
X1740
X1742
X1743
X1747

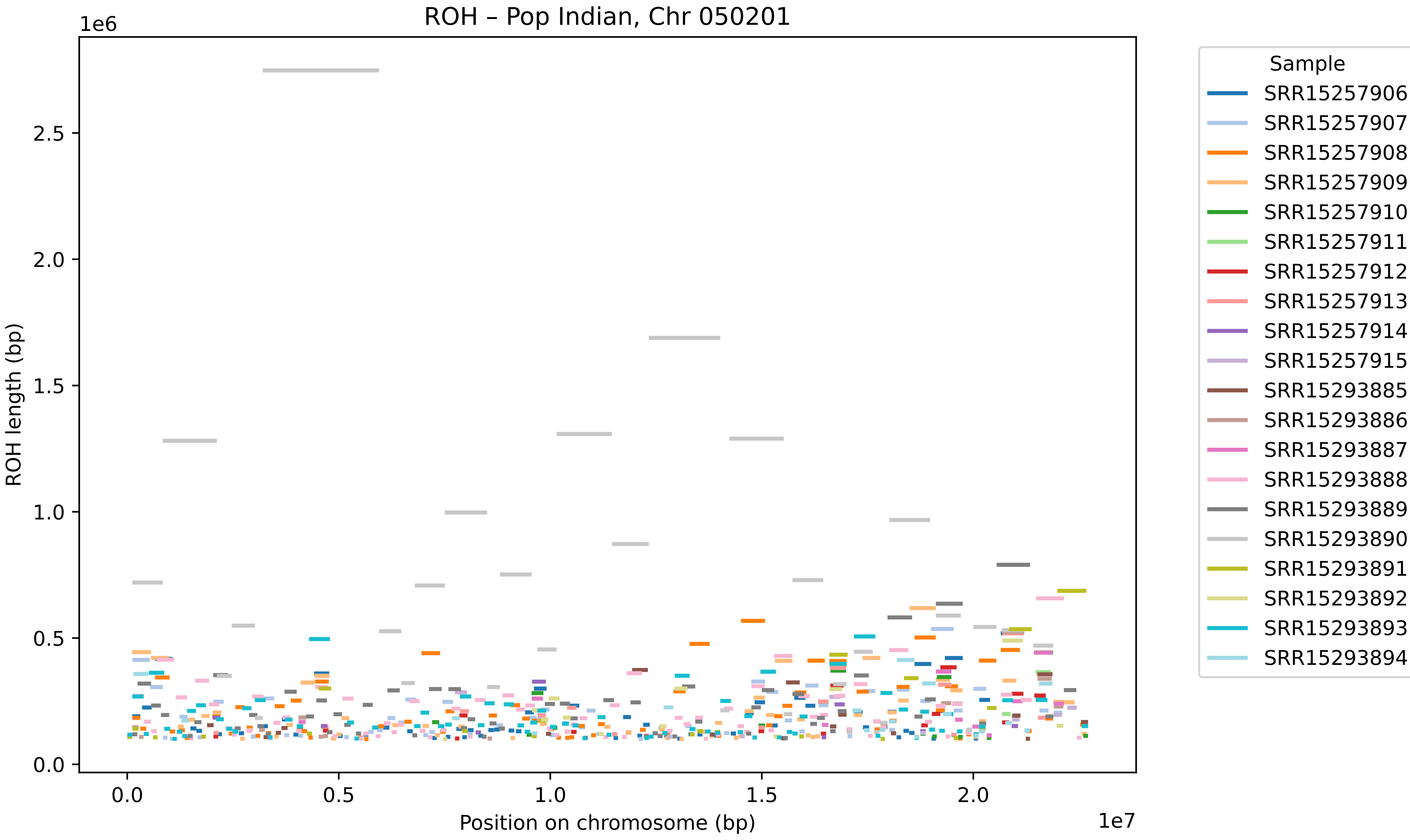

ROH – Pop Indian, Chr 050201
1e6
2.5
2.0
1.5
1.0
0.5
0.0
ROH length (bp)
0.0
0.5
1.0
1.5
2.0
1e7
Position on chromosome (bp)
Sample
SRR15257906
SRR15257907
SRR15257908
SRR15257909
SRR15257910
SRR15257911
SRR15257912
SRR15257913
SRR15257914
SRR15257915
SRR15293885
SRR15293886
SRR15293887
SRR15293888
SRR15293889
SRR15293890
SRR15293891
SRR15293892
SRR15293893
SRR15293894

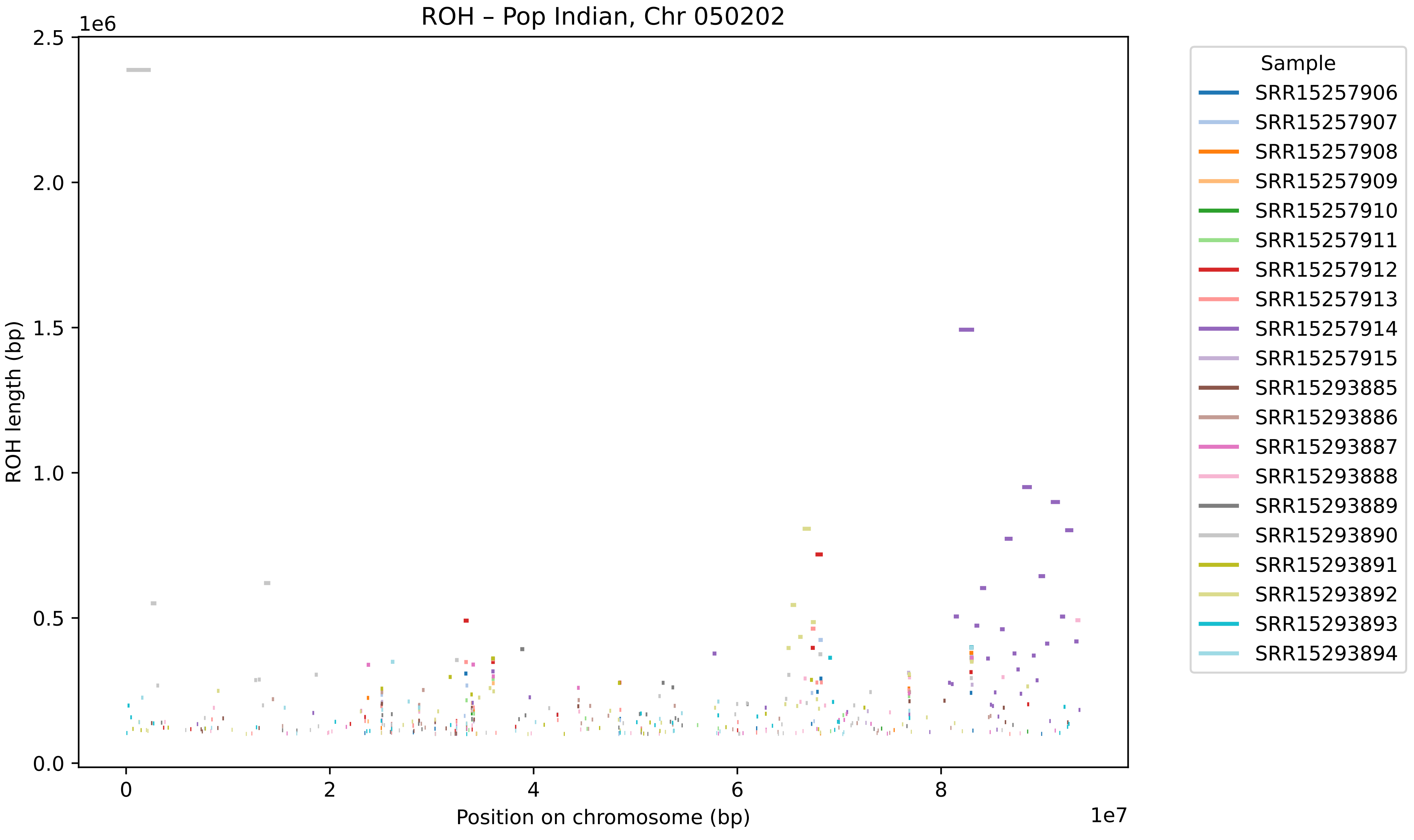
ROH – Pop Indian, Chr 050202
1e6
2.5
2.0
1.5
1.0
0.5
0.0
ROH length (bp)
0
2
4
6
8
1e7
Position on chromosome (bp)
Sample
SRR15257906
SRR15257907
SRR15257908
SRR15257909
SRR15257910
SRR15257911
SRR15257912
SRR15257913
SRR15257914
SRR15257915
SRR15293885
SRR15293886
SRR15293887
SRR15293888
SRR15293889
SRR15293890
SRR15293891
SRR15293892
SRR15293893
SRR15293894

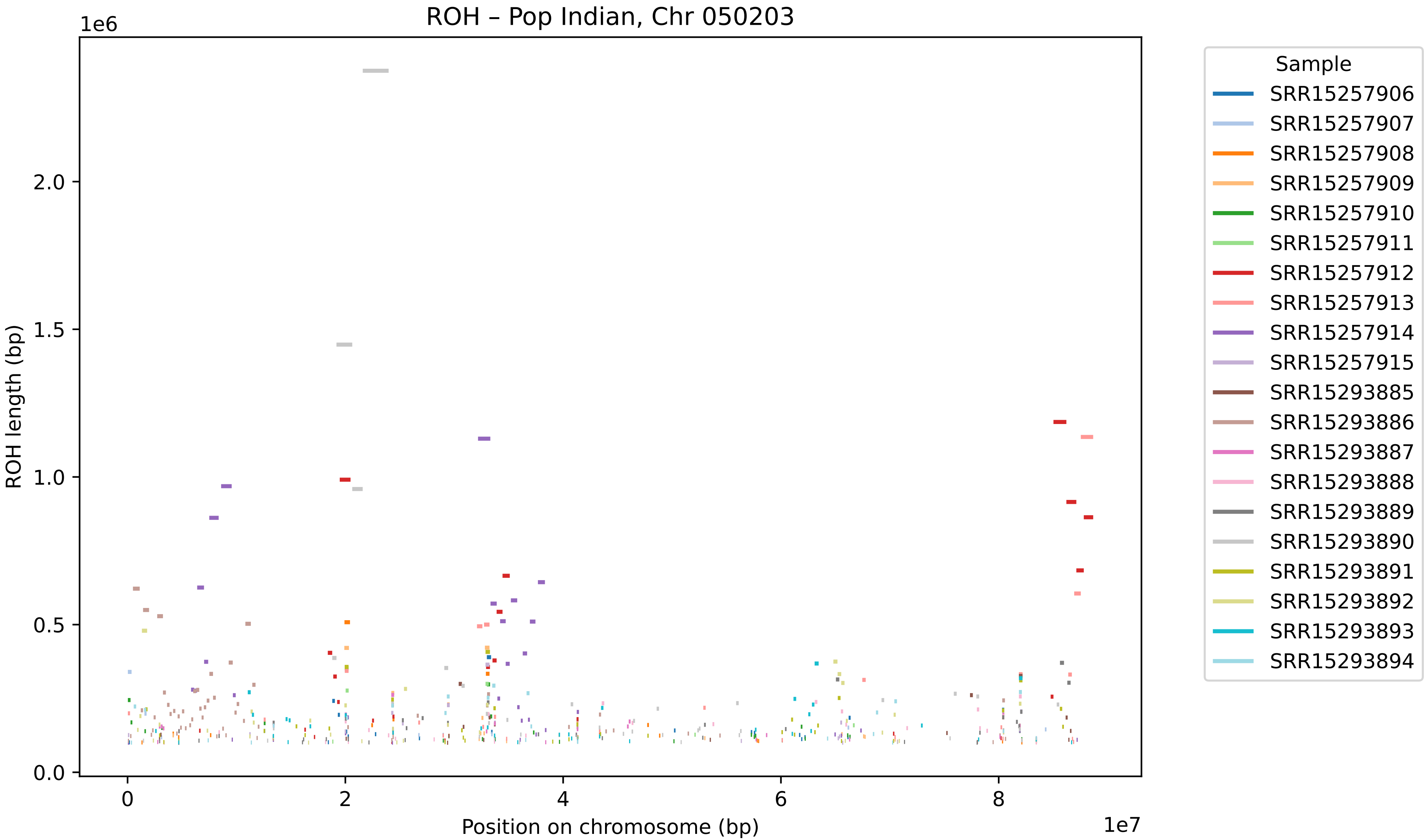
ROH – Pop Indian, Chr 050203
1e6
ROH length (bp)
0.0
0.5
1.0
1.5
2.0
Position on chromosome (bp)
1e7
0
2
4
6
8
Sample
SRR15257906
SRR15257907
SRR15257908
SRR15257909
SRR15257910
SRR15257911
SRR15257912
SRR15257913
SRR15257914
SRR15257915
SRR15293885
SRR15293886
SRR15293887
SRR15293888
SRR15293889
SRR15293890
SRR15293891
SRR15293892
SRR15293893
SRR15293894

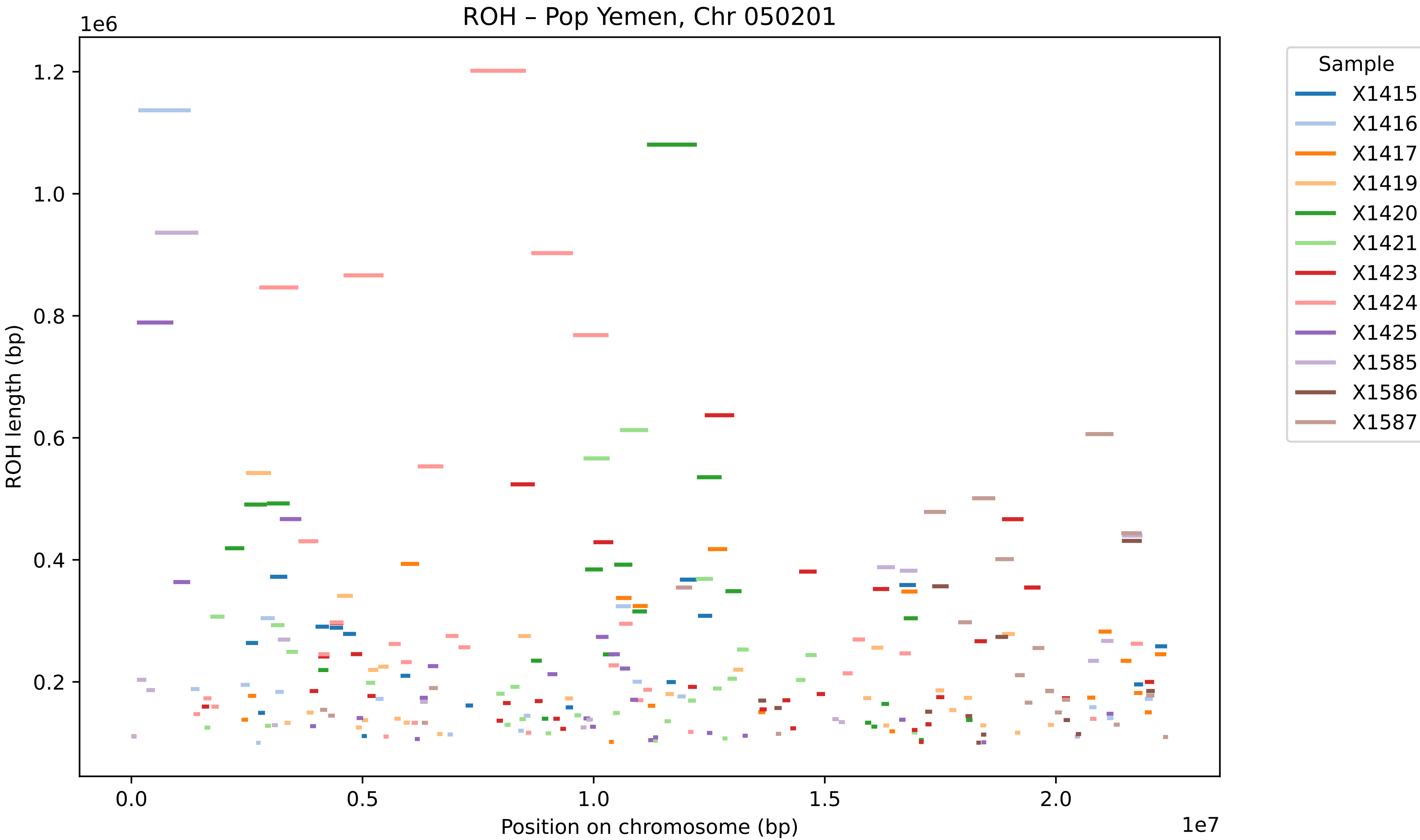
ROH – Pop Yemen, Chr 050201
1e6
1.2
1.0
0.8
0.6
0.4
0.2
ROH length (bp)
0.0
0.5
1.0
1.5
2.0
1e7
Position on chromosome (bp)
Sample
X1415
X1416
X1417
X1419
X1420
X1421
X1423
X1424
X1425
X1585
X1586
X1587

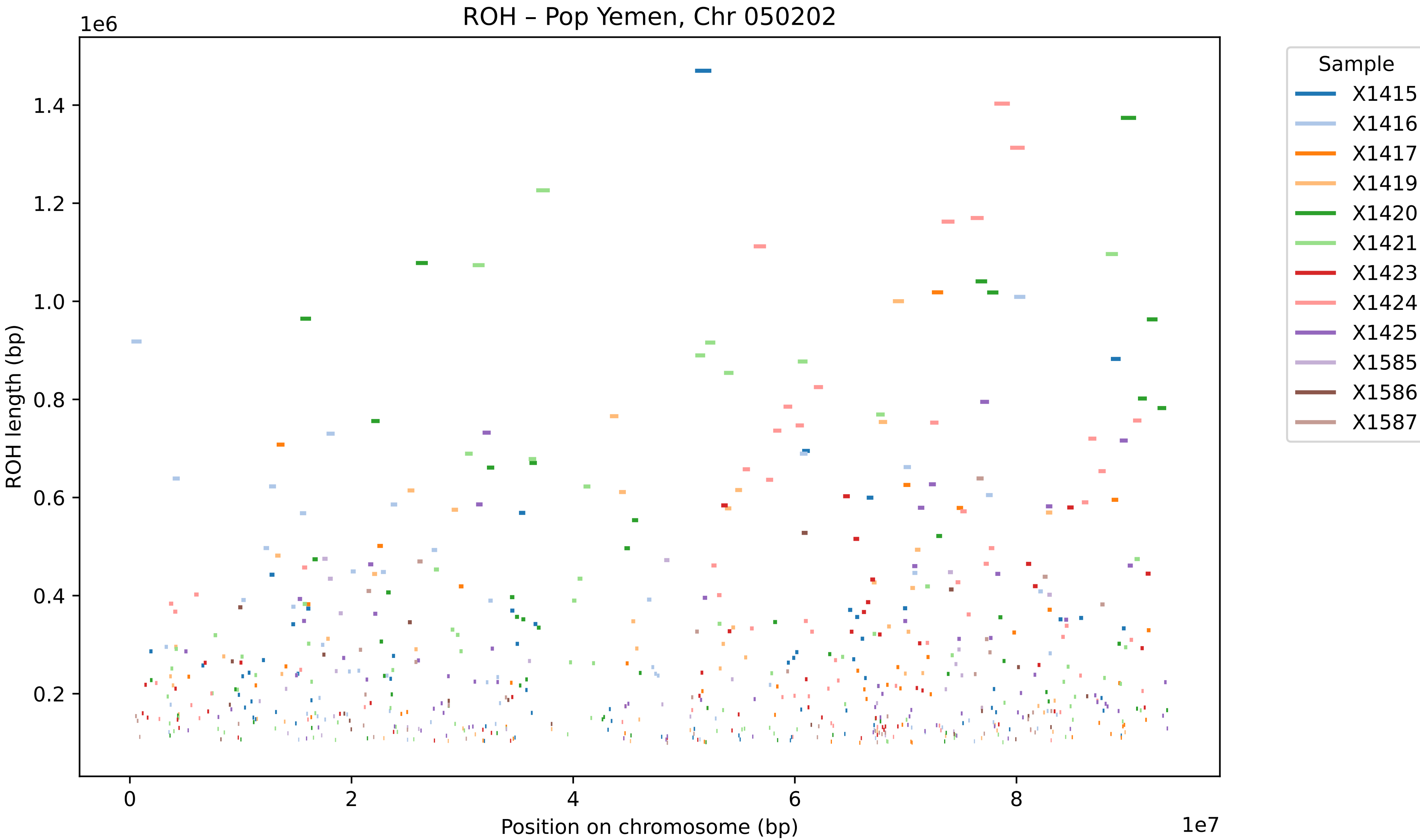
ROH – Pop Yemen, Chr 050202
1e6
1.4
1.2
1.0
0.8
0.6
0.4
0.2
ROH length (bp)
0
2
4
6
8
1e7
Position on chromosome (bp)
Sample
X1415
X1416
X1417
X1419
X1420
X1421
X1423
X1424
X1425
X1585
X1586
X1587

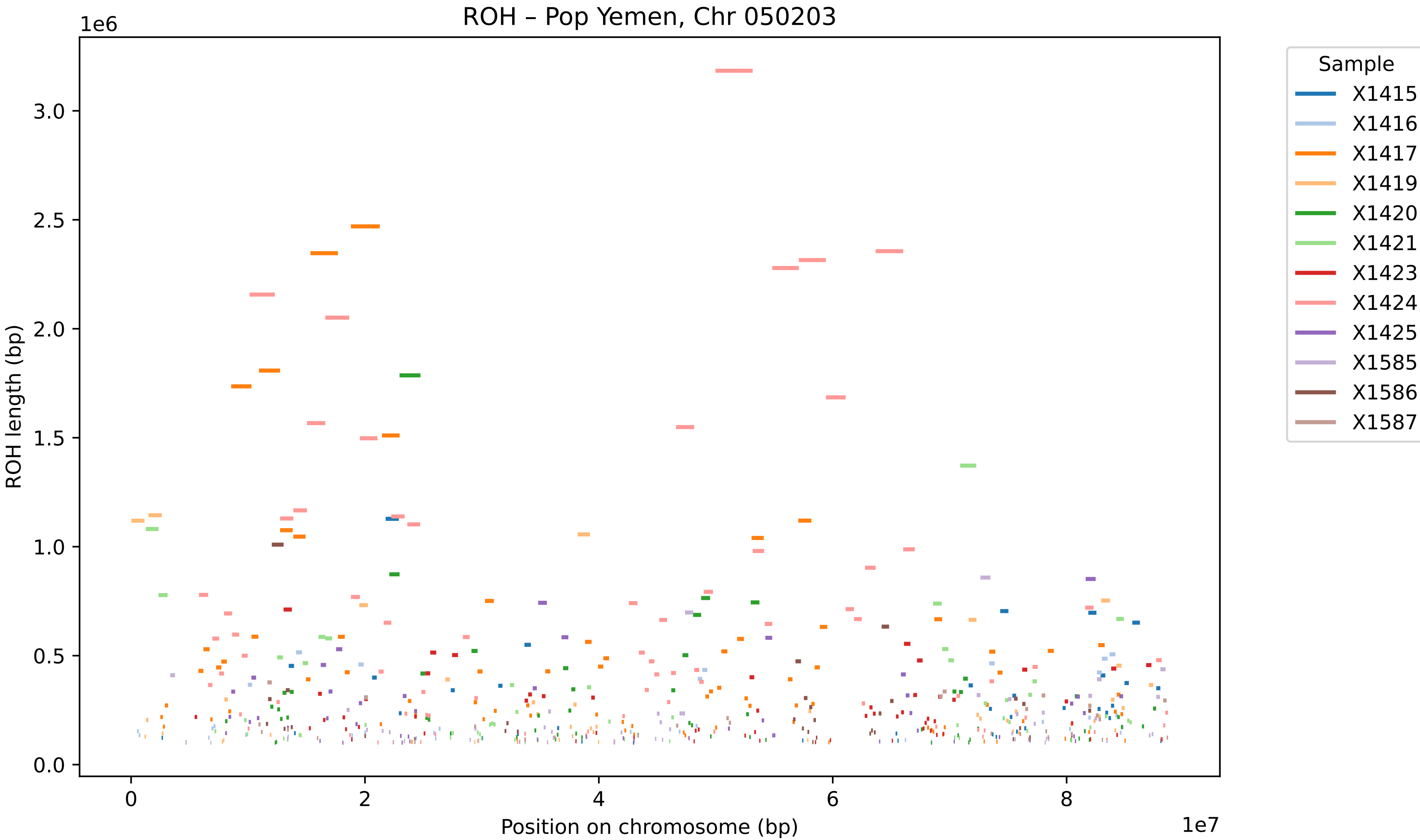

ROH – Pop Yemen, Chr 050203
1e6
ROH length (bp)
0.0
0.5
1.0
1.5
2.0
2.5
3.0
Position on chromosome (bp)
0
2
4
6
8
1e7
Sample
X1415
X1416
X1417
X1419
X1420
X1421
X1423
X1424
X1425
X1585
X1586
X1587

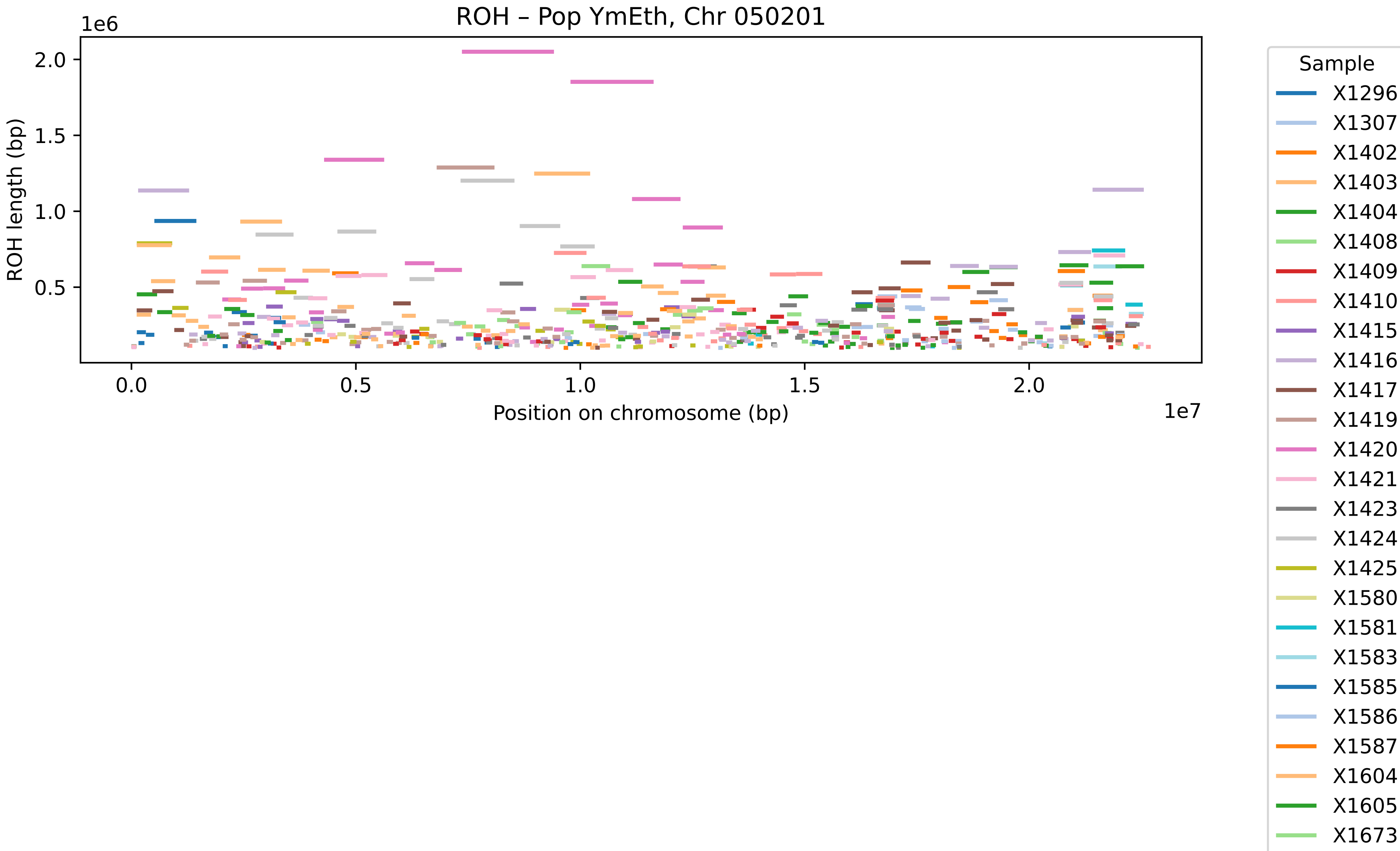
ROH – Pop YmEth, Chr 050201
1e6
ROH length (bp)
2.0
1.5
1.0
0.5
0.0
0.5
1.0
1.5
2.0
1e7
Position on chromosome (bp)
Sample
X1296
X1307
X1402
X1403
X1404
X1408
X1409
X1410
X1415
X1416
X1417
X1419
X1420
X1421
X1423
X1424
X1425
X1580
X1581
X1583
X1585
X1586
X1587
X1604
X1605
X1673

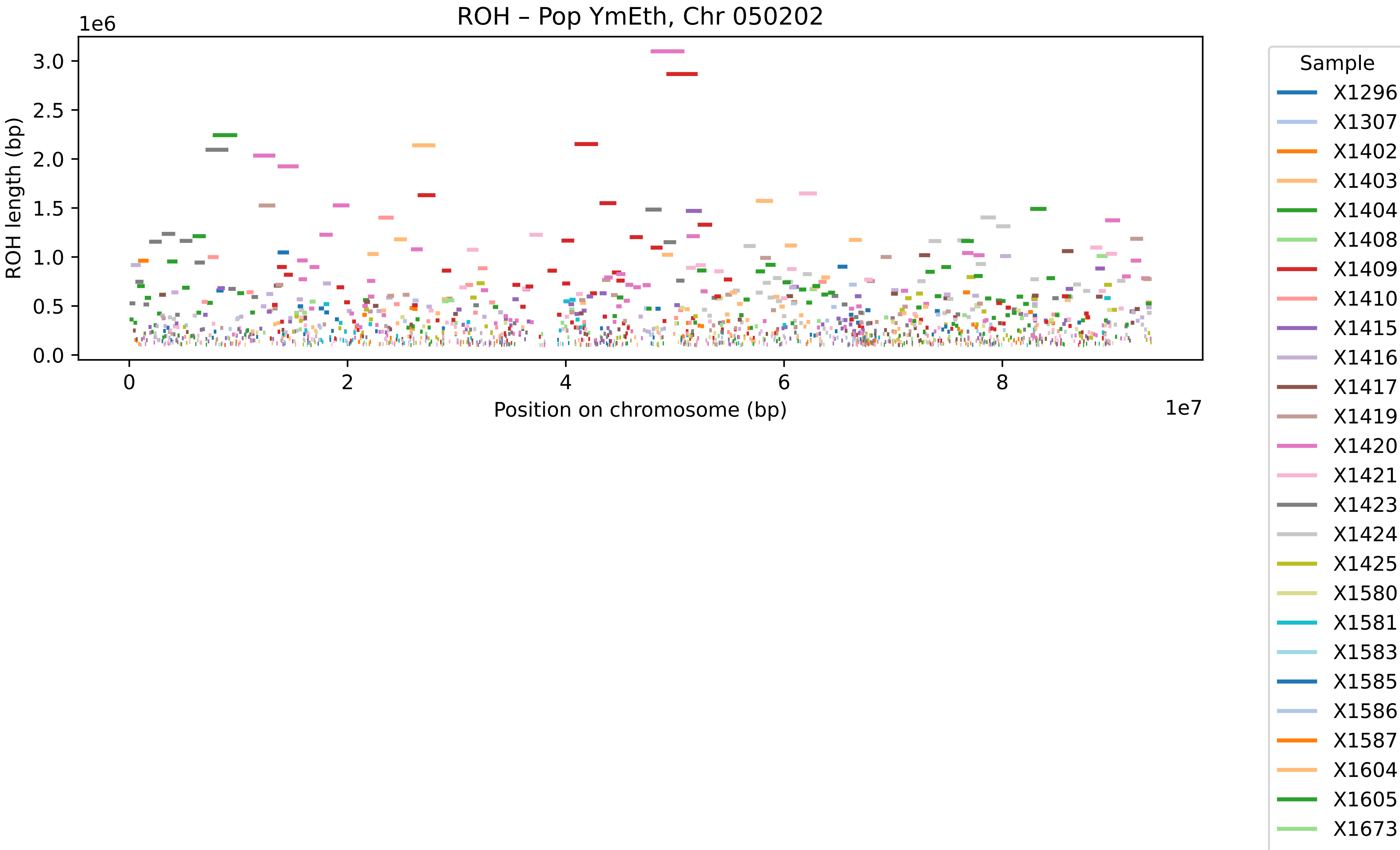

ROH – Pop YmEth, Chr 050202
1e6
3.0
2.5
2.0
1.5
1.0
0.5
0.0
ROH length (bp)
0
2
4
6
8
1e7
Position on chromosome (bp)
Sample
X1296
X1307
X1402
X1403
X1404
X1408
X1409
X1410
X1415
X1416
X1417
X1419
X1420
X1421
X1423
X1424
X1425
X1580
X1581
X1583
X1585
X1586
X1587
X1604
X1605
X1673

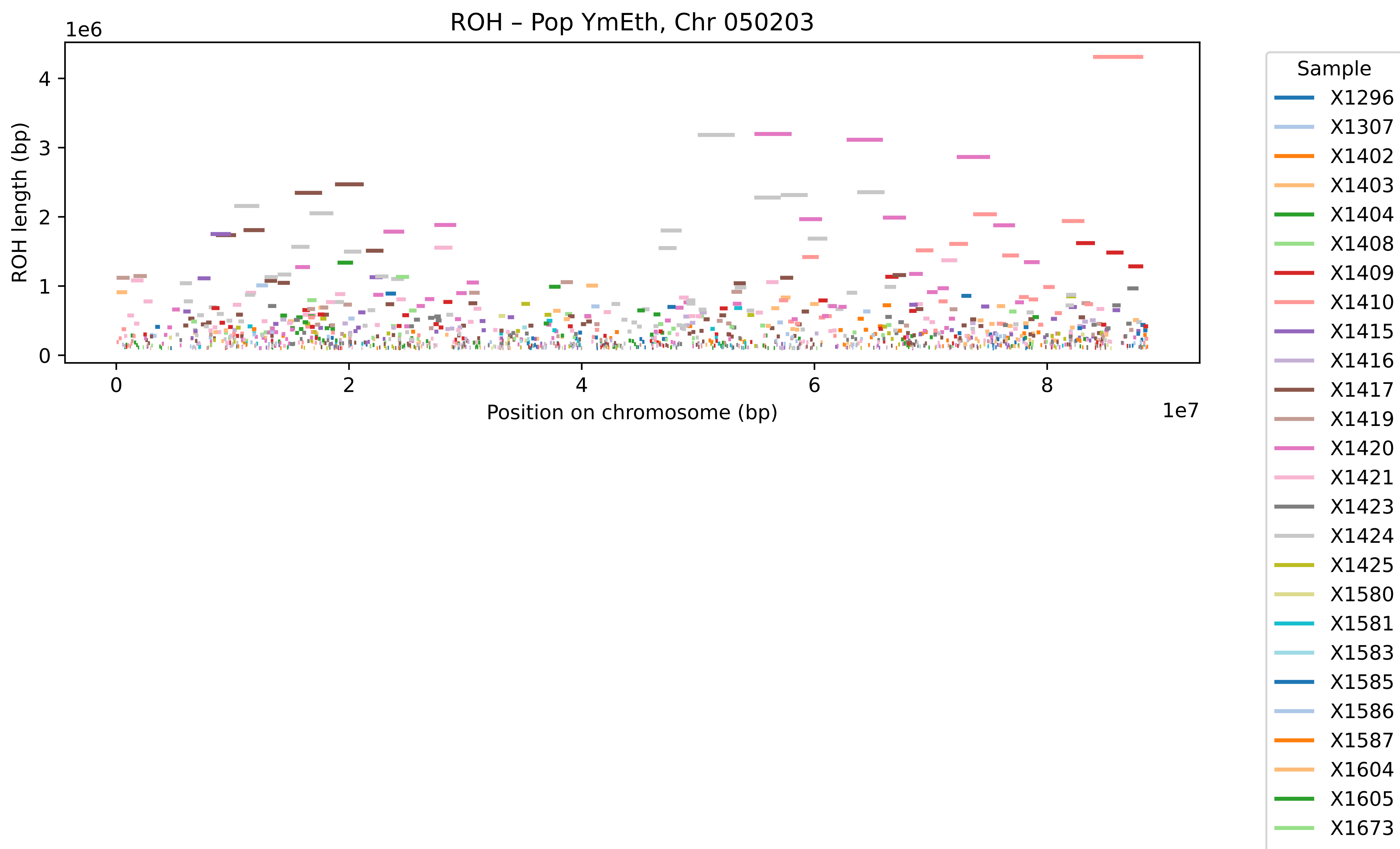

ROH – Pop YmEth, Chr 050203
1e6
4
3
2
1
0
ROH length (bp)
0
2
4
6
8
1e7
Position on chromosome (bp)
Sample
X1296
X1307
X1402
X1403
X1404
X1408
X1409
X1410
X1415
X1416
X1417
X1419
X1420
X1421
X1423
X1424
X1425
X1580
X1581
X1583
X1585
X1586
X1587
X1604
X1605
X1673
