# Supplementary material for: Multiple genomic approaches reveal geographic structure and local selection signals in invasive Anopheles stephensi from the Horn of Africa and Yemen
Source: BMC Genomics. 2026 Jul 7;27:802. doi: 10.1186/s12864-026-13079-x (PMC13625371; doi:10.1186/s12864-026-13079-x)

WGS ADMIXTURE plots with Indian samples (K = 3 – 10)

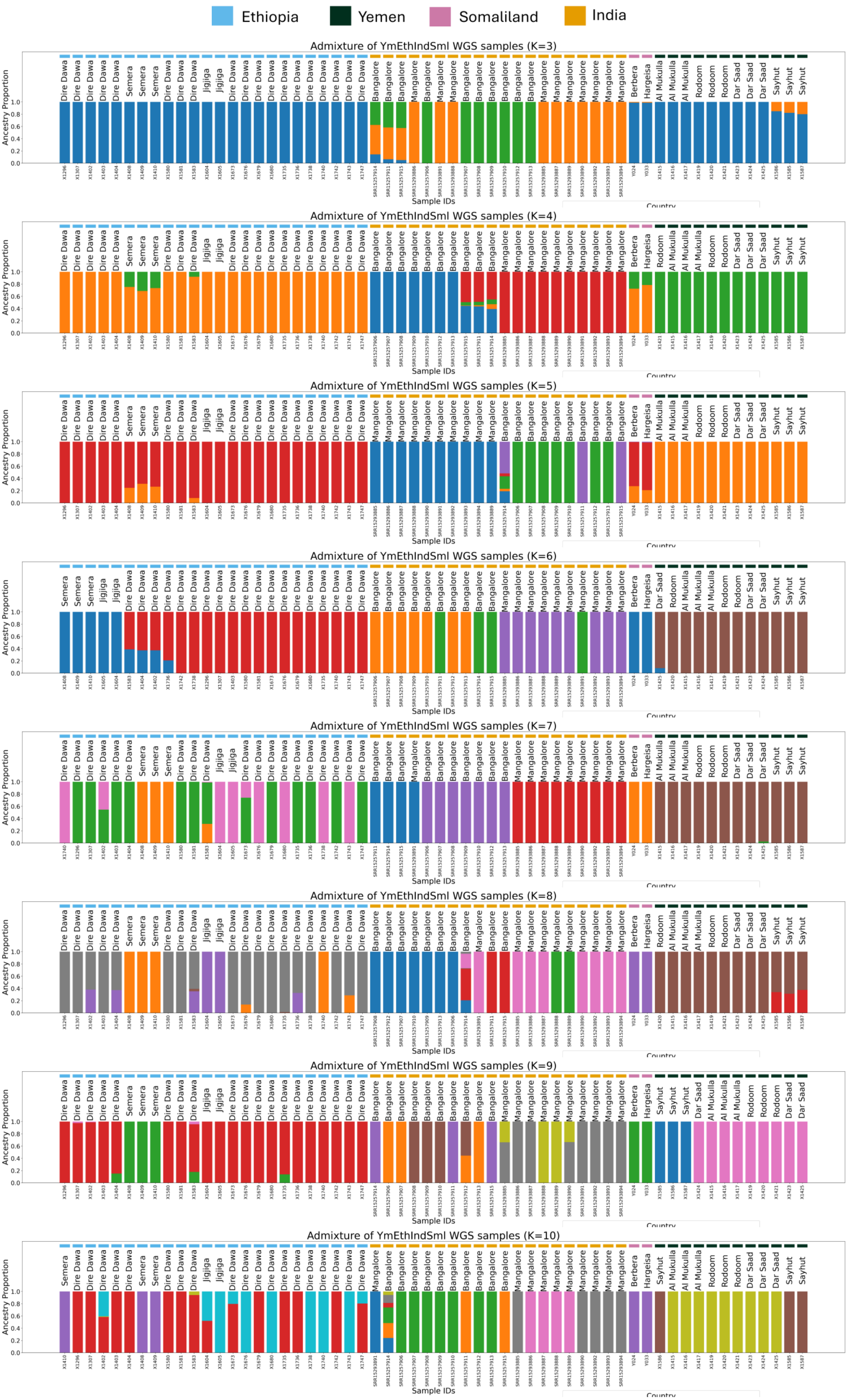

WGS ADMIXTURE plots without Indian samples (K = 3 – 10)

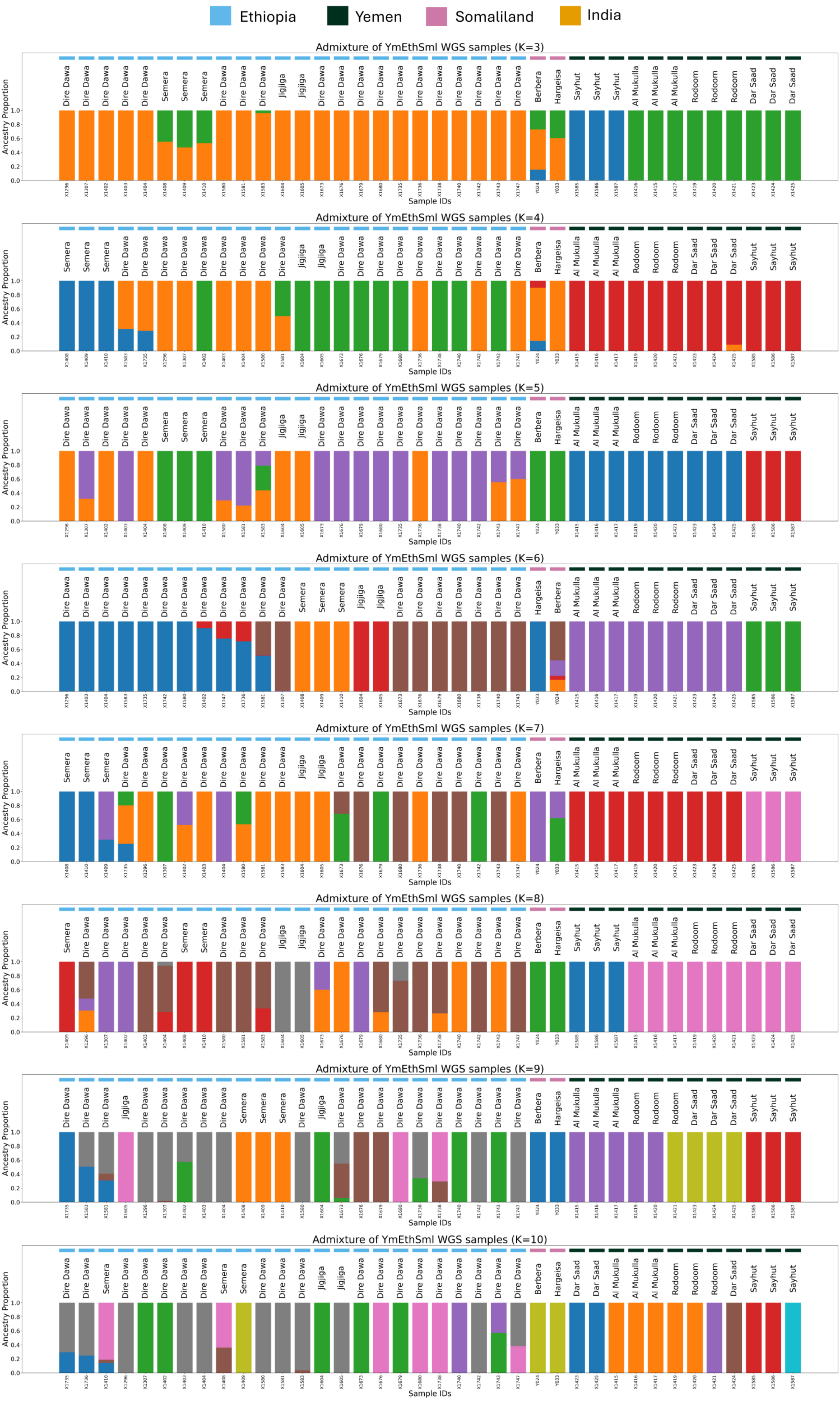

Supplement: Supplementary file 2 [file 12864_2026_13079_MOESM2_ESM.pdf]
