# Supplementary material for: Multiple genomic approaches reveal geographic structure and local selection signals in invasive Anopheles stephensi from the Horn of Africa and Yemen
Source: BMC Genomics. 2026 Jul 7;27:802. doi: 10.1186/s12864-026-13079-x (PMC13625371; doi:10.1186/s12864-026-13079-x)
Supplement: Supplementary file 3 [file 12864_2026_13079_MOESM3_ESM.pdf]

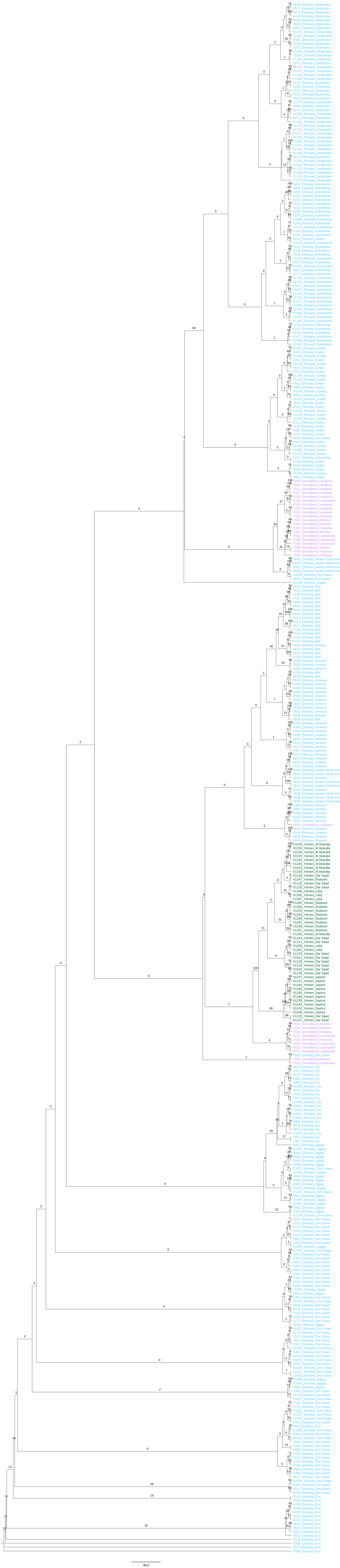
40.0
