# Supplementary material for: Multiple genomic approaches reveal geographic structure and local selection signals in invasive Anopheles stephensi from the Horn of Africa and Yemen
Source: BMC Genomics. 2026 Jul 7;27:802. doi: 10.1186/s12864-026-13079-x (PMC13625371; doi:10.1186/s12864-026-13079-x)
Supplement: Supplementary file 4 [file 12864_2026_13079_MOESM5_ESM.pdf]

# Supplementary Methods

## Sample collection, sequencing and variant calling of whole genome sequencing

*Anopheles stephensi* mosquitos were collected between 2018 –2022 from the sites mentioned in Figure 1from Ethiopia and Yemen. Mosquitoes were collected as wild caught adults and lab reared adults from larvae. The samples from two laboratory-reared Indian colonies (Mangalore and Bangalore) were utilized from a previously published study (20). Field-collected individuals were preserved on silica and transported to the laboratory in United States of America for DNA extraction. High-quality genomic DNA was extracted using QIAGEN DNeasy® Blood & Tissue Kit, following the manufacturers protocol and quantified using Thermo Scientific™ Invitrogen™ Nanodrop™ One Spectrophotometer. Whole-genome sequencing was conducted using the Illumina NovaSeq X Plus Series (PE150) platform, generating 150 bp paired-end reads. Quality control of raw reads was performed using FastQC(21), followed by adapter trimming and low-quality base removal using Trimmomatic(23).

### Variant Calling and Filtering of whole genome sequence data

Reads were aligned to the *An. stephensi* reference genome (UCI) using BWA-MEM, and duplicate reads were marked using Picard tools in Genome Analysis Tool Kit (GATK) (22, 24, 33). Variant calling was performed using the GATK HaplotypeCaller, followed by joint genotyping across all samples using GenotypeGVCFs. SNPs were filtered based on read depth, mapping quality, strand bias, and other quality metrics using GATK's best practices (24). Only bi-allelic SNPs with a minimum depth of 10 and a minor allele frequency (MAF) > 0.1 were retained. Separate VCFs were generated for whole genome, individual chromosomes, and mitochondrial genomes.

## Sample collection, sequencing and variant calling of mitochondrial genomes

Adult mosquitoes were collected from various locations across three countries (Yemen, Ethiopia, and Somaliland) during the summers of 2018, 2022 and 2023. The mosquitoes were captured with the aid of a mouth aspirator from outdoor manholes, artificial resting sites such as large water containers, abandoned ponds and discarded car tires. Following collection, mosquitoes were subjected to morphological screening for species identification. A total of 26 mosquitoes, identified as *Anopheles stephensi*, were selected for further screening.

### DNA extraction, amplification and sequencing

Genomic DNA was extracted from the whole body of each of the selected mosquito species using the DNeasy Blood & Tissue kit (Qiagen, Valencia, CA, USA). Upon

extraction, the species identification (ID) of each mosquito was confirmed through the amplification and sequencing of two genetic loci namely the Internal transcribed spacer region 2 locus (*ITS2*) and the mitochondrial cytochrome c oxidase subunit 1 (*COI*). The PCR mixture included 1x Promega HotStart Master Mix (Promega, Madison, WI) and 0.5 mM of primers for both *ITS2* and *COI*, and 1 µl of the extracted DNA template. A 650 bp region of the *ITS2* gene was amplified using universal primers (51). The thermal cycling conditions were: 95 °C for 1 min, followed by 30 cycles of 95 °C for 30 sec, 48 °C for 30 sec, and 72 °C for 1 min; with a final extension at 72 °C for 10 min. Similarly, the *COI* gene was amplified using universal primers (3). The thermal cycling protocol was performed as follows: 95 °C for 1 min, 30 cycles of 95 °C for 30 sec, 48 °C for 30 sec, and 72 °C for 1 min; and a final extension of 72 °C for 10 min. Both *ITS2* and *COI* genes were successfully amplified in all samples.

Two approaches were employed to obtain the complete mitogenome of *An. stephensi*. The first approach involved the use of Primer3Plus and Primer-Blast programs, utilizing the reference mitogenome of *An. stephensi* as a template. These programs were used to design primers targeting specific regions of the *An. stephensi* mitogenome. A set of 18 primer pairs had been previously designed to amplify the mitogenome (52). However, gaps were observed in the assembled mitogenomes of some samples. To address these gaps, we designed an additional three primer pairs.

PCR amplifications were performed in a 25 µl reaction mixture consisting of 1 µl of DNA template, 12.5 µl of 1x GoTAQ Hot Start Master Mix, 1 µl of 10 µM forward and reverse primers, and 8.5 µl of dd$H_2O$. The thermal cycling conditions were: 94 °C for 5 min, followed by 35 cycles of 94 °C for 1 min, 48–56 °C (depending on the primer pair) for 45 sec, and 68 °C for 1 min, with a final extension at 72 °C for 10 min. All PCR products were separated by electrophoresis on a 1% agarose gel and subsequently sent for sequencing. The PCR products were subjected to Sanger sequencing to obtain raw nucleotide chromatograms. The chromatograms were trimmed to remove low-quality bases, and converted fasta sequences which were aligned to the *An. stephensi* reference mitogenome using CodonCode Aligner.

The second approach involved extracting mitogenomes from the whole genome sequencing (WGS) data. WGS reads were mapped to the *An. stephensi* reference mitogenome (GenBank accession number: NC_028223.1) to obtain complete mitogenomes, leveraging the depth of coverage provided by WGS datasets. This method served as an alternative to PCR-based amplification, enabling the recovery of mitogenomes directly from genomic data.

## Sample collection and sequencing for ddRAD data

Samples originated from five locations where *An. stephensi* was found across western and southern Yemen and collected in 2021 and 2022. Briefly, larvae were collected using dipping methods from different larval breeding habitats per site as part of *Aedes*

and *Anopheles* surveillance and reared to adults in field insectaries. Female adult *Anopheles* were morphologically identified at the species level using the updated Afrotropical mosquitoes key (10). The specimens morphologically identified as *An. stephensi* were preserved with silica gel and a subsample of specimens was sent to Baylor University for molecular analysis and DNA extractions.

### DNA extraction, ddRAD-seq library preparation, and SNP genotyping

Genomic DNA was extracted from adult *An. stephensi* mosquitoes using Qiagen DNeasy Blood and Tissue kit (Qiagen, CA). DNA quality was assessed on a 1% agarose gel to ensure high molecular weight bands and quantified using Nanodrop One Spectrophotometer (Thermo Fisher Scientific Inc., MA, USA) to ensure a minimum concentration of 20 ng/ul. ddRAD-seq library preparation followed protocols outlined in Samake et al. in 2023 (10). In short, genomic DNA was enzymatically fragmented using SbfI and EcoRI restriction enzymes, and Illumina TruSeq compatible barcodes ligated for future demultiplexing. Libraries were quantified, pooled in equimolar amounts, and the multiplexed library sent to Novogene (Novogene CO., Ltd., Sacramento, CA, USA) for 150-bp, single-end chemistry sequencing on an Illumina HiSeq X.

Raw Illumina reads were demultiplexed, processed and SNP genotyped using the computational pipeline described in Lavretsky et al. 2020 and following steps outlined in Samake et al. 2023 (13, 53). Briefly, individual sample reads were parsed based on barcode/index sequences. Sequences were trimmed using Trimmomatic  and reads with a PHRED score of <30 were discarded to ensure only high-quality sequences were retained (23). The remaining quality reads were aligned to the *An. stephensi* reference genome (Accession PRJNA661063) using the Burrows Wheeler Aligner (bwa) (22, 33).

### ddRAD and mitochondrial genome variant calling

The variants were called from the aligned ".bam" files using the mpileup option in BCFtools (version 1.17) program (54). Variants were normalized and filtered with parameters specifically optimized for mitochondrial DNA to get the highest quality variant call respective to the reference mitogenomes. These parameters included a minimum allele frequency threshold of 1% to capture variants and Phred-scaled quality scores greater than 30 to ensure the reliability of the variant calls. Additionally, a read depth threshold of 10 was applied to filter out low-confidence variants and focus on high-quality data. After variant calling, BCFtools was used to refine the variant set further by excluding potential sequencing artifacts and low-quality calls, ensuring that only robust variants were retained. The mitogenome sequences were extracted from the VCF files using SAMtools program (version 1.18) and consensus option in BCFtools program. Extracted sequences were saved in FASTA format for the downstream analysis. The sequences were aligned using Clustal and Muscle programs (55, 56).
